# Culturable Fungi from Urban Soils in China I: Description of 10 New Taxa

**DOI:** 10.1128/Spectrum.00867-21

**Published:** 2021-10-06

**Authors:** Zhi-Yuan Zhang, Qiu-Yu Shao, Xin Li, Wan-Hao Chen, Jian-Dong Liang, Yan-Feng Han, Jian-Zhong Huang, Zong-Qi Liang

**Affiliations:** a Institute of Fungus Resources, Department of Ecology, College of Life Sciences, Guizhou University, Guiyang, China; b Department of Microbiology, Guiyang College of Traditional Chinese Medicine, Guiyang, China; c Engineering Research Center of Industrial Microbiology, Ministry of Education, Fujian Normal University, Fuzhou, China; University of Texas at San Antonio

**Keywords:** new taxa, keratinophilic fungi, *Thelebolales*, soil fungi, *Zongqia*, hair baiting technique

## Abstract

An investigation of members of the soil keratinophilic fungi community in China resulted in the identification of one new monotypic genus, *Zongqia*, and 10 new species, 2 of which are affiliated with *Solomyces*, 1 with the new genus *Zongqia*, 4 with *Pseudogymnoascus*, and 3 with *Scedosporium*. These novel taxa form an independent lineage distinct from other species, based on morphological and multilocus phylogenetic analyses. Descriptions, illustrations, and notes are provided for each taxon. These new taxa of the soil keratinophilic fungi add to the increasing number of fungi known from China, and it is now evident that numerous novel taxa are waiting to be described.

**IMPORTANCE** Keratinophilic fungi are a group that can degrade and utilize keratin-rich material. It is also because of this ability that many taxa can cause infections in animals or humans but remain poorly studied. In this study, we reported a novel genus and 10 novel species, 7 novel species belonging to the order *Thelebolales* and 3 to the genus *Scedosporium*, based on multilocus phylogenetic analyses combined with morphological characteristics. Our study significantly updates the taxonomy of *Thelebolales* and *Scedosporium* and enhances our understanding of this group of the keratin-degrading fungal community. The findings also encourage future studies on the artificially constructed keratin-degrading microbial consortia.

## INTRODUCTION

Soil microbes are the richest component of terrestrial biodiversity, and among them, soil fungi play a major role in the ecosystem processes. To date, many studies have explored fungi in ocean, caves, forests (especially pristine rainforests), extreme environments, volcanoes, mountains, deserts, freshwater aquatic systems, lakes, grasslands, indoor environments, and many other habitats (1), and they have found that fungi in different habitats have very high species diversity. At the same time, many new fungal taxa have been reported, and they have shown potential high value in the industries of agriculture and medicine. However, as global urbanization continues to expand (2, 3), urban soil fungi, which are closely related to human health, have not been systematically investigated although they are a focal area for ecological and environmental issues. China has diverse urban soil types, diverse habitats, rapid urbanization, and high population mobility. Investigating the diversity of soil fungi in different cities in China will provide scientific data for understanding their ecological functions and maintaining public health safety and will enable the isolation of many new resources with potential applications.

The enrichment culture method using different substrates can often screen for the specific fungal consortium, so this method is often used for the isolation of fungal taxa with specific physiological functions. The distribution of keratinophilic fungi, as a special fungal consortium that can degrade and utilize keratin-rich materials, is greatly influenced by the activities of humans and animals, and the presence of such fungus is high in areas where humans and animals are frequently active, especially in urban parks, hospitals, and school campuses (4–7). According to the habitat, keratinophilic fungi can be broadly classified into three eco-types, anthropophilic, zoophilic, and geophilic species, and are mostly pathogenic or potentially pathogenic fungi. For human health and safety, their distribution should attract the attention of governments and scientists. Keratinophilic fungi have been reported in soils of different habitats in different geographic regions of the world, so the investigation of keratinophilic fungi has epidemiological significance (8).

Since the report of the degradable keratin of Onygena equina (9), new taxa of keratinophilic fungi and their applications have been reported. Keratinophilic fungi involve a large number of taxa belonging to several orders, families, and genera, including mainly dermatophytes and some saprophytic fungi, such as some species of *Arthrodermataceae* and *Onygenaceae* in the order *Onygenales* (10) and some members of the genera *Geomyces* and *Pseudogymnoascus* in the order *Thelebolales* (11). In addition, they contain a large number of common taxa, such as some species of the genera *Aspergillus*, *Penicillium*, and *Trichoderma* (12, 13). In the years since we investigated the members of keratin-degrading fungal communities in Chinese soils, several new taxa have been identified and reported (14–22). Here, we introduce one new genus, *Zongqia* (*Thelebolales* genera *incertae sedis*, *Thelebolales*), and 10 new species, 2 of which are affiliated with *Solomyces*, 1 with the new genus *Zongqia*, 4 with *Pseudogymnoascus*, and 3 with *Scedosporium*.

## RESULTS

In this study, the internal transcribed spacer (ITS) regions of all isolates were sequenced, and all ITS sequences obtained were BLASTn searched in NCBI and assigned to potential genera and species. Then, strains belonging to *Thelebolales* and *Scedosporium* were screened and tested for further identification through morphological characterization and phylogenetic analyses.

### Molecular phylogeny.

The first concatenated alignment (including *Pseudogymnoascus* and its related taxa) consisted of 2,806 nucleotides, including inserted gaps (ITS: 430 bp, large subunit ribosomal DNA [LSU]: 790 bp, minichromosomal maintenance protein 7 [*MCM7*]: 485 bp, RNA polymerase II subunit 2 [*RPB2*]: 467 bp, and elongation factor 1 alpha [*EF1A*]: 634 bp). The second concatenated data set (mainly involving the genera of *Thelebolales*) included 1,208 nucleotides, including inserted gaps (ITS: 433 bp; LSU: 775 bp). The third concatenated matrix (including *Scedosporium* and its related taxa) contained 964 nucleotides, including inserted gaps (ITS: 544 bp; beta-tubulin [*BT2*]: 420 bp). The best-fit evolutionary models of ML and BI analyses of each locus are listed in Table 1. The tree topology from both maximum likelihood (ML) and Bayesian interference (BI) analyses was almost identical.

**TABLE 1:** The best-fit evolutionary models in our phylogenetic analyses

Data set	Method	Model					
		ITS	*BT2*	LSU	*MCM7*	*RPB2*	*EF1A*
First	ML	TIM2e + I + G4		GTR + F + I	TVMe + I + G4	K2P + I + G4	SYM + R3
	BI	SYM + I + G4		TR + F + I	SYM + I + G4	K2P + I + G4	SYM + I + G4
Second	ML	SYM + R3		TIM + F + I + G4			
	BI	SYM + I + G4		GTR + F + I + G4			
Third	ML	TIM3 + F + R3	HKY + F + R2				
	BI	HKY + F + I + G4	HKY + F + G4				

**FIG 1:**
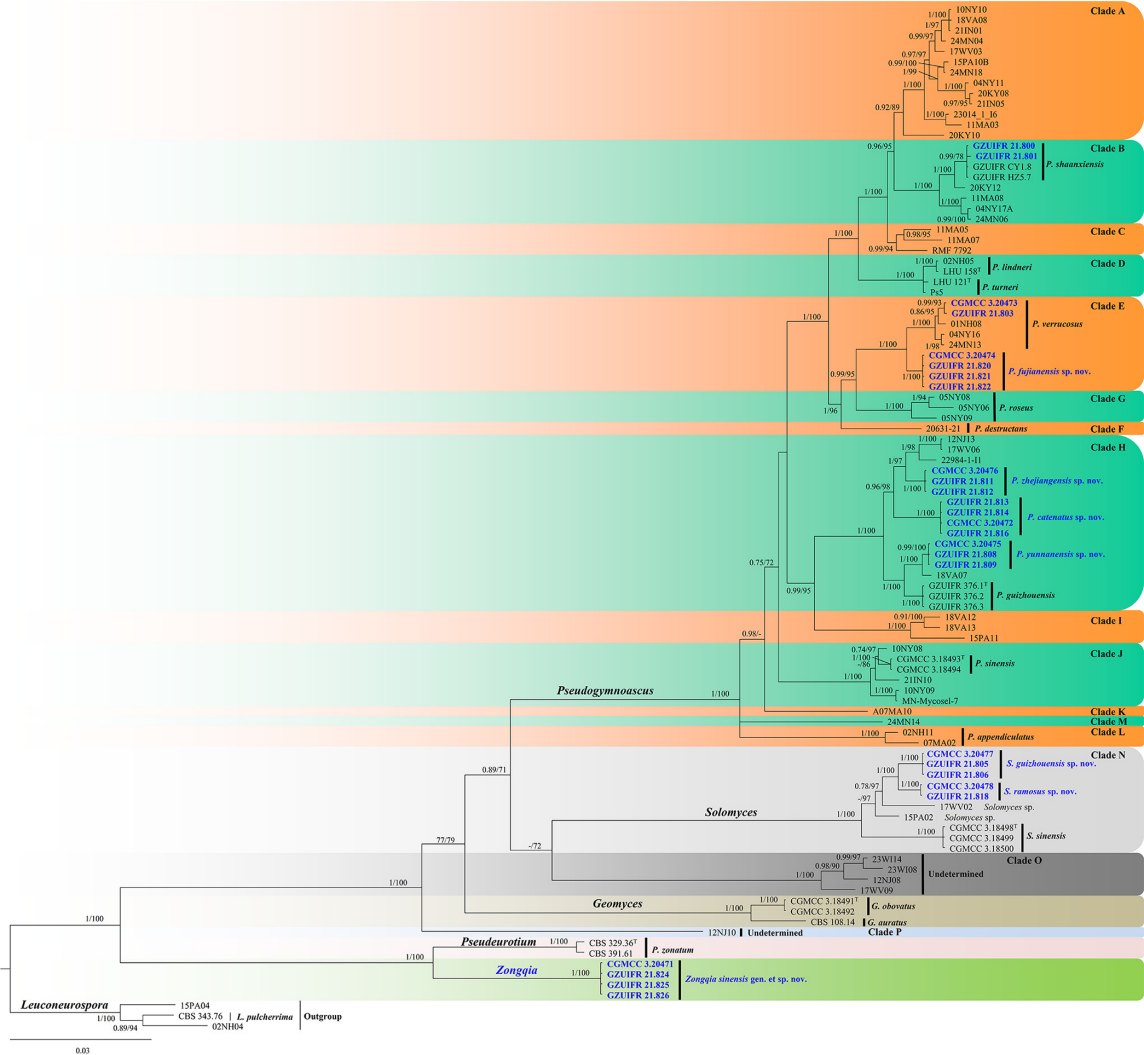
Bayesian inference strict consensus tree illustrating the phylogeny of new taxa and related species in *Thelebolales* based on a five-loci (ITS, LSU, *MCM7*, *RPB2*, *EF1A*) concatenated data set. Branches are labeled with Bayesian posterior probabilities of >0.70 and maximum likelihood bootstrap values of >70%. The new taxa and strains are in bold and blue. Clade names follow previous studies (21, 24).

In the first phylogenetic tree (Fig. 1), the clades formed by each genus and by undetermined taxa had a high support rate: *Pseudogymnoascus* (1 posterior probability [PP]/100% bootstrap support [BS]), *Solomyces* (1 PP/100% BS), undetermined (clade O, 1 PP/100% BS), *Geomyces* (1 PP/100% BS), *Pseudeurotium* (1 PP/100% BS), and *Zongqia* gen. nov. (1 PP/100% BS). Our new species is divided into three genera. Eighteen of our new strains belong to three clades of genus *Pseudogymnoascus*, five are contained in genus *Solomyces*, and the remaining four are located within the new genus *Zongqia*.

**FIG 2:**
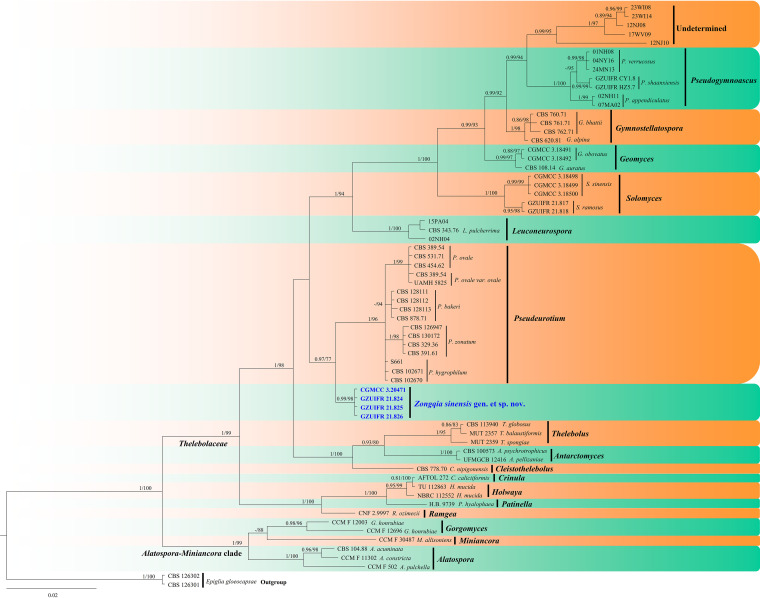
Bayesian inference strict consensus tree illustrating the phylogeny of genera in *Thelebolales* based on a two-loci (ITS and LSU) concatenated data set. Branches are labeled with Bayesian posterior probabilities of >0.70 and maximum likelihood bootstrap values of >70%. The new taxa and strains are in bold and blue.

In the second phylogenetic tree (Fig. 2), each genus clusters into a monophyletic clade. The new genus *Zongqia* forms a well-supported (0.99 PP/98% BS) clade separated from other genera in *Thelebolales*.

**FIG 3:**
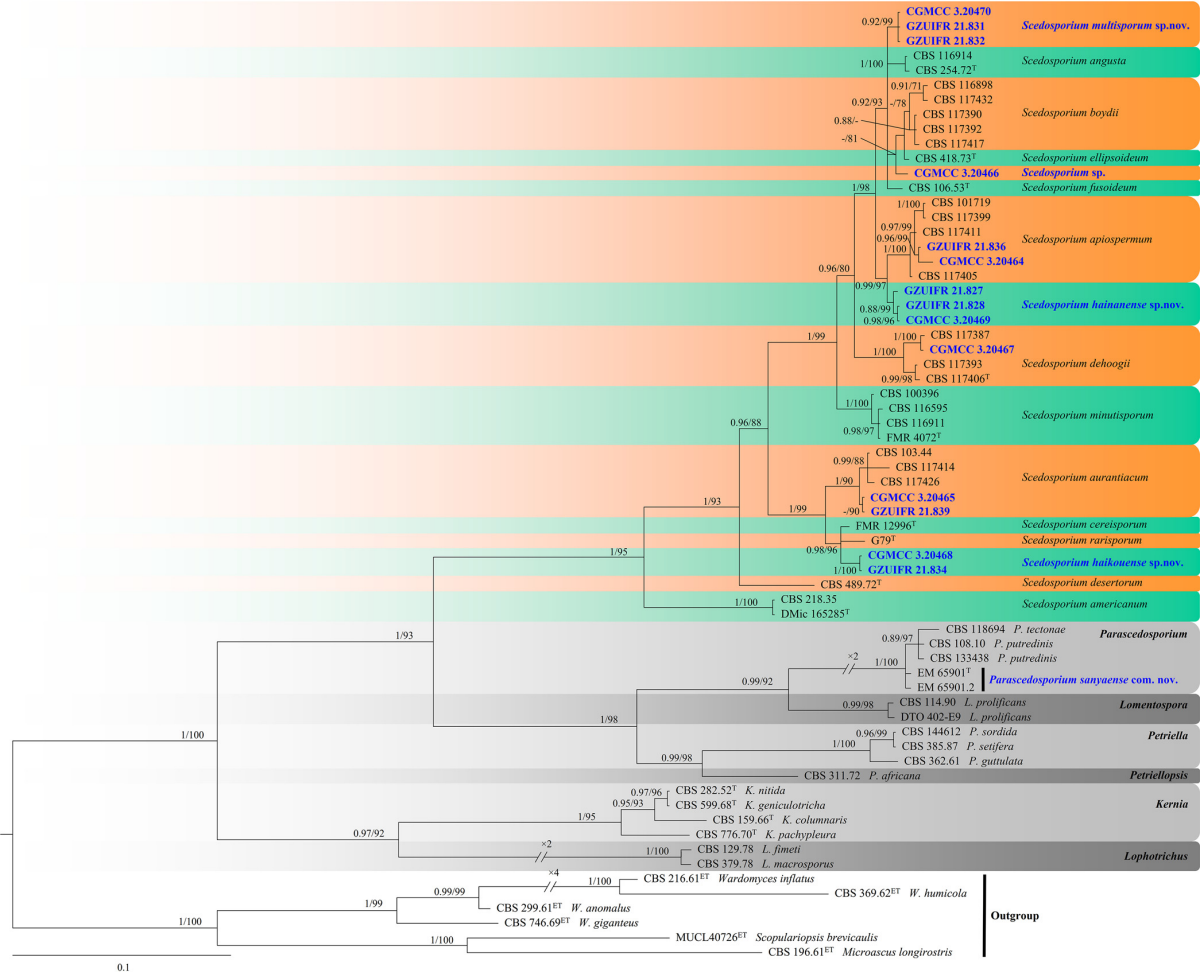
Phylogeny of *Scedosporium* and related species generated by BI analyses based on combined two-loci (ITS and *BT2*) sequences. Branches are labeled with Bayesian posterior probabilities of >0.70 and maximum likelihood bootstrap of >70%, respectively. New species and strains are indicated in bold and blue.

In the third phylogenetic tree (Fig. 3), the clades formed by each genus had a high support rate: *Scedosporium* (1 PP/95% BS), *Parascedosporium* (1 PP/100% BS), *Lomentospora* (0.99 PP/98% BS), *Petriella* (1 PP/100% BS), *Kernia* (1 PP/95% BS), and *Lophotrichus* (1 PP/100% BS). Our new species is nested in *Scedosporium*, and our strains are spread into five well-supported main clades, representing the species Scedosporium hunanense sp. nov. (0.92 PP/99% BS), Scedosporium apiospermum (1 PP/100% BS), Scedosporium hainanense sp. nov. (0.88 PP/99% BS), Scedosporium aurantiacum (1 PP/90% BS), and Scedosporium haikouense sp. nov. (1 PP/100% BS), except for CGMCC3.20466, which is associated with the species Scedosporium boydii and Scedosporium ellipsoideum.

## TAXONOMY

**FIG 4:**
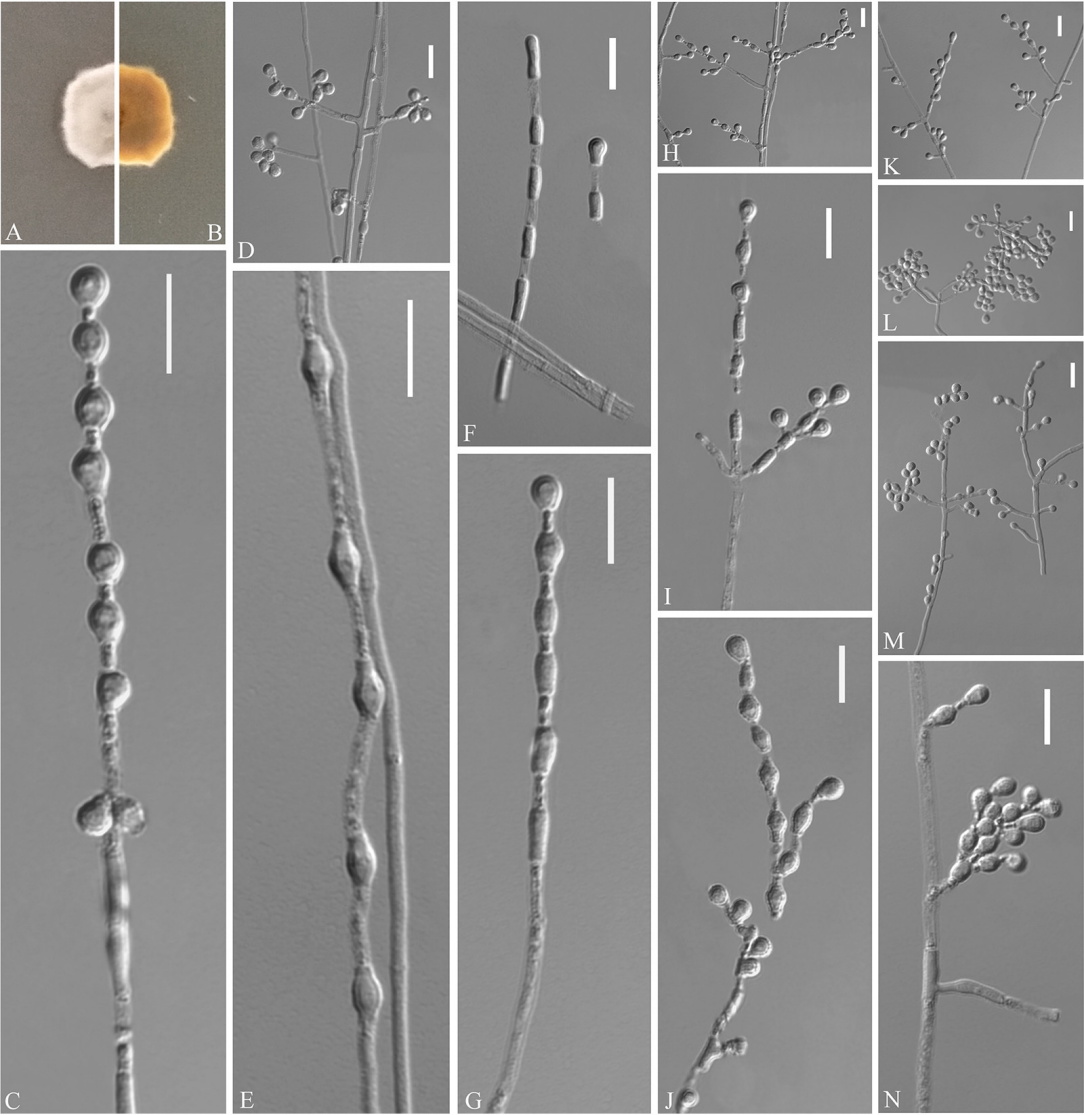
*Pseudogymnoascus catenatus* (from ex-holotype CGMCC 3.20472). (A, B) Upper and reverse views of culture on PDA 14 days after inoculation; (C, E, G, I, J) intercalary conidia; (D, H, K to N) conidiophores and conidia; (F) arthroconidia. Scale bars (C to N), 10 μm.

Pseudogymnoascus catenatus Zhang, Han, and Liang, sp. nov. (Fig. 4). MycoBank number: MB 840436. Etymology: referring to the catenation of its intercalary conidia. Diagnosis: similar to Pseudogymnoascus verrucosus but differs in obovoid conidia and intercalary conidia. Type: China, Fujian Province, Wuyishan City, Lie Ning Park, 27.758010N, 118.034403E, isolated from green belt soil, 18 August 2019, Z.Y. Zhang. (Holotype HMAS 350322, stored in a metabolically inactive state; ex-holotype culture CGMCC 3.20472 = GZUIFR 21.815, *ibid*., GZUIFR 21.816.) GenBank: MZ444080, MZ444081 (ITS); MZ444107, MZ444108 (LSU); MZ490762, MZ490763 (*MCM7*); MZ488545, MZ488546 (*RPB2*); MZ488522, MZ488523 (translation elongation factor [*TEF*]).

Description. Sexual morph: not observed. Asexual morph: colonies on peptone-dextrose agar (PDA) slowly growing, attaining 6 to 10 mm diameter after 14 days at 25°C, velvety, short and fluffy, margins irregular, light gray to white, absent pigment and exudates; reverse brown. No growth at 37°C. Hyphae hyaline, branched, septate, smooth, 1 to 3 μm wide. Racquet hyphae absent. Conidiophores abundant, frequent branches, at acute angles, often 1 to 2 verticillate with 1 to 4 branches per whorl, secondary and tertiary branches can still branch again. Conidia abundant, normally borne terminally on verticillate branches or borne laterally and solitary on short protrusions or short side branches; subhyaline to hyaline, smooth-walled or rough; obovoid, sometimes subglobose, 3.0 to 6.0 by 3.0 to 4.0 μm (*n* = 50). Intercalary conidia are borne on the verticillate hyphae or hyphae, solitary or 1 to 6 in chains, smooth-walled or rough, obovoid, subglobose, fusiform, drum-shaped, truncated at both ends, 3.5 to 6.5 by 3.0 to 4.5 μm (*n* = 50), cylindrical, barrel-shaped, truncated at both ends, 5.5 to 6.5 by 2.5 to 3.5 μm (*n* = 50). Arthroconidia hyaline, cylindrical, sometimes obovoid, 3.0 to 6.0 by 2.0 to 3.5 μm (*n* = 50).

Substrate: soil. Distribution: Wuyishan City, Fujian Province; Ningbo City, Zhejiang Province, China. Material examined: China, Zhejiang Province, Ningbo City, Moon Lake, 29.870001N, 121.544021E, isolated from green belt soil, 16 August 2019, Z.Y. Zhang, GZUIFR 21.813, *ibid*., GZUIFR 21.814. GenBank: MZ444078, MZ444079 (ITS); MZ444105, MZ444106 (LSU); MZ490760, MZ490761 (*MCM7*); MZ488543, MZ488544 (*RPB2*); MZ488520, MZ488521 (*TEF*).

Notes. Morphologically, *Pseudogymnoascus catenatus* is similar to P. verrucosus in having arthroconidia but is clearly distinguished by the obovoid conidia and intercalary conidia (23). Phylogenetically, four isolates of P. catenatus formed a single clade separate from other species in *Pseudogymnoascus* (Fig. 1), which indicates that they are distinct species.

**FIG 5:**
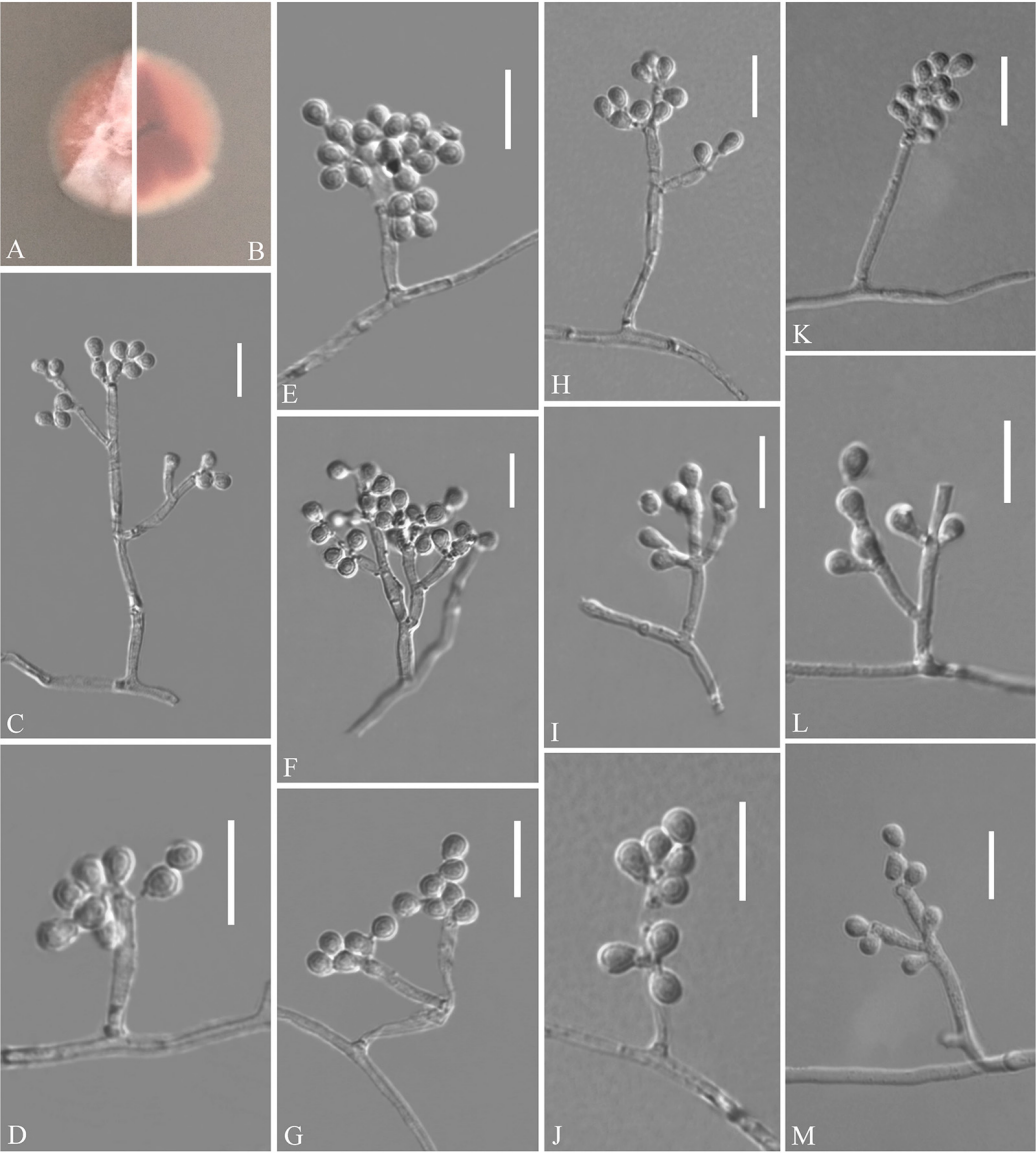
*Pseudogymnoascus fujianensis* (from ex-holotype CGMCC 3.20474). (A, B) Upper and reverse views of culture on PDA 14 days after inoculation; (C to M) conidiophores, conidia, and intercalary conidia. Scale bars (C to M), 10 μm.

Pseudogymnoascus fujianensis Zhang, Han, and Liang, sp. nov. (Fig. 5). MycoBank number: MB 840437. Etymology: refers to the region from which the fungus was isolated. Diagnosis: similar to *P. verrucosus*, Pseudogymnoascus roseu, and Pseudogymnoascus destructans but differs in the presence of intercalary conidia and the absence of arthroconidia. Type: China, Fujian Province, Wuyishan City, Lie Ning Park, 27.758545N, 118.034134E, isolated from green belt soil, 18 August 2019, Z.Y. Zhang. (Holotype HMAS 350324, stored in a metabolically inactive state; ex-holotype culture CGMCC 3.20474 = GZUIFR 21.819, *ibid*., GZUIFR 21.820.) GenBank: MZ444084, MZ444085 (ITS); MZ444111, MZ444112 (LSU); MZ490766, MZ490767 (*MCM7*); MZ488549, MZ488550 (*RPB2*); MZ488526, MZ488527 (*TEF*).

Description. Sexual morph: not observed. Asexual morph: colonies on PDA attaining 19 to 20 mm diameter after 14 days at 25°C, flat, flocculent, sectorization, margin identified, white to pink, absent pigment and exudates; reverse brown. No growth at 37°C. Hyphae hyaline, branched, septate, smooth-walled, 0.5 to 3.5 μm wide. Racquet hyphae absent. Conidiophores abundant, branches, at acute angles, irregular, acyclic arrangement. Conidia abundant, mostly terminal or lateral, sessile or borne on hyphae, short protrusions or side branches; solitary, fasciation, or 2 in chains; hyaline, smooth-walled; obovoid, 2.5 to 5.5 by 2.5 to 4.0 μm (*n* = 50). Intercalary conidia abundant, normally chained with terminal conidia; solitary, smooth-walled or rough; obovoid, sometimes drum-shaped, 2.5 to 5.0 by 2.5 to 3.5 μm (*n* = 50).

Substrate: soil. Distribution: Wuyishan City, Fujian Province, China. Material examined: China, Fujian Province, Wuyishan City, Wuyi University, 27.728722N, 118.002862E, isolated from green belt soil, 18 August 2019, Z.Y. Zhang, GZUIFR 21.821, *ibid*., GZUIFR 21.822. GenBank: MZ444086, MZ444087 (ITS); MZ444113, MZ444114 (LSU); MZ490768, MZ490769 (*MCM7*); MZ488551, MZ488552 (*RPB2*); MZ488528, MZ488529 (*TEF*).

Notes. Morphological and phylogenetic analyses (Fig. 1) support our four strains as new species of *Pseudogymnoascus fujianensis*. P. fujianensis is phylogenetically closely related to *P. verrucosus*, *P. roseu*, and P. destructans. However, *P. fujianensis* is distinguished from other species of *Pseudogymnoascus* by the presence of intercalary conidia and the absence of arthroconidia (23–26).

**FIG 6:**
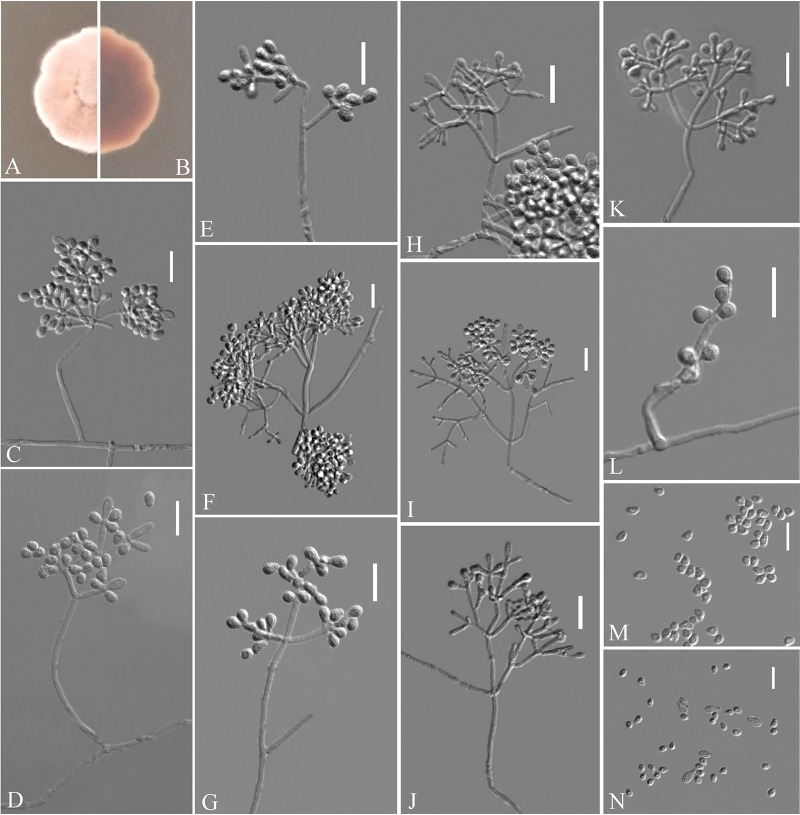
*Pseudogymnoascus yunnanensis* (from ex-holotype CGMCC 3.20475). (A, B) Upper and reverse views of culture on PDA 14 days after inoculation; (C to L) conidiophores and conidia; (M to N) conidia. Scale bars (C to N), 10 μm.

Pseudogymnoascus yunnanensis Zhang, Han, and Liang, sp. nov. (Fig. 6). MycoBank number: MB 840438. Etymology: refers to the region from which the fungus was isolated. Diagnosis: similar to Pseudogymnoas lindneri, Pseudogymnoas turneri, and Pseudogymnoas guizhouensis but differs in the clavate, fusiform with basal scars terminal conidia, and reniform, fusiform, truncated at both ends of intercalary conidia. Type: China, Yunnan Province, Dali City, Dali Bai Autonomous Prefecture People’s Hospital, 25.578478N, 100.222121E, isolated from green belt soil, 3 September 2019, Z.Y. Zhang. (Holotype HMAS 350320, stored in a metabolically inactive state; ex-holotype culture CGMCC 3.20475 = GZUIFR 21.807, *ibid*., GZUIFR 21.808.) GenBank: MZ444072, MZ444073 (ITS); MZ444099, MZ444100 (LSU); MZ490754, MZ490755 (*MCM7*); MZ488537, MZ488538 (*RPB2*); MZ488514, MZ488515 (*TEF*).

Description. Sexual morph: not observed. Asexual morph: colonies on PDA attaining 23 to 25 mm diameter after 14 days at 25°C, velvety, powdery, margin identified, locally indented, pink, white at the edge, absent pigment and exudates; reverse brown. No growth at 37°C. Hyphae hyaline, branched, septate, smooth-walled, 1 to 3 μm wide. Racquet hyphae absent. Conidiophores abundant, frequent branches, at acute angles, often 2 to 3 verticillate with 1 to 4 branches per whorl, secondary and tertiary branches can still branch again. Conidia abundant, normally borne terminally on verticillate branches, or borne laterally and solitary on short protrusions or short side branches; subhyaline to hyaline, smooth-walled or echinulate; obovoid, subglobose to globose, sometimes pyriform, 2.5 to 4.5 by 2.5 to 3.5 μm (*n* = 50); sometimes terminal conidia clavate, fusiform with basal scars, 6.5 to 9.0 by 2.5 to 4.5 μm (*n* = 50). Intercalary conidia are borne on the outer branches of the hyphae or verticillate hyphae, solitary or two in chains, smooth-walled or rough, reniform and fusiform truncate at both ends, 2.5 to 5.5 by 2.5 to 4.0 μm (*n* = 50).

Substrate: soil. Distribution: Dali City, Yunnan Province, China. Material examined: China, Yunnan Province, Dali City, Dali University, 25.674141N, 100.154757E, isolated from green belt soil, 2 September 2019, Z.Y. Zhang, GZUIFR 21.809. GenBank: MZ444074 (ITS); MZ444101 (LSU); MZ490756 (*MCM7*); MZ488539 (*RPB2*); MZ488516 (*TEF*).

Notes. Morphologically, *Pseudogymnoascus yunnanensis* is similar to *P. lindneri*, *P. turneri*, and *P. guizhouensis* in having obovoid, globose conidia (27). However, *P. yunnanensis* can be distinguished from *P. lindneri* and *P. turneri* by the presence of its clavate, fusiform with basal scars terminal conidia and no observed sexual morph. *P. yunnanensis* differs from *P. guizhouensis* because it is reniform, fusiform, and truncated at both ends of intercalary conidia (22). Phylogenetically, three isolates of *P. yunnanensis* constitute a strongly supported subclade, sister to *P. guizhouensis* with high support values (Fig. 1), but they can be easily distinguished.

**FIG 7:**
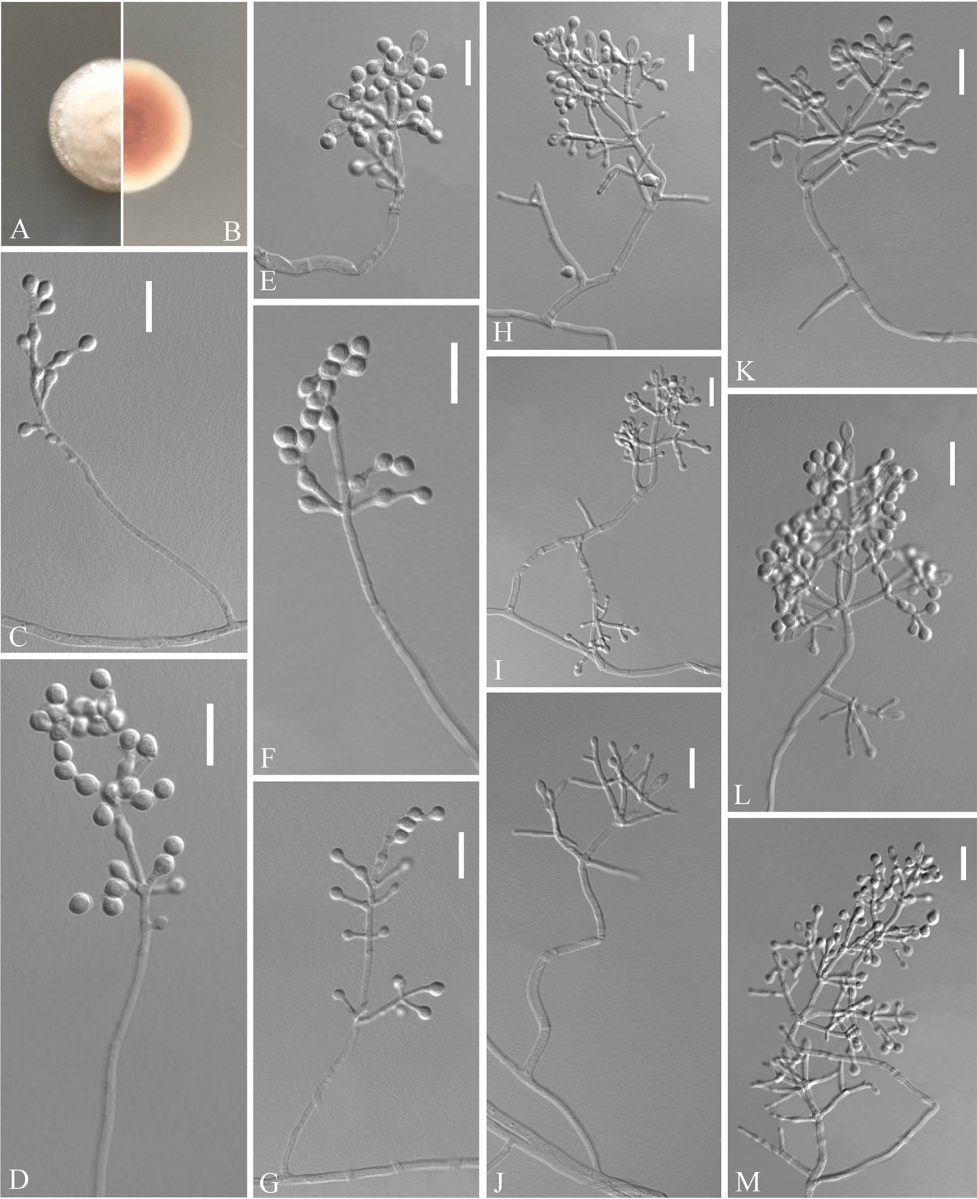
*Pseudogymnoascus zhejiangensis* (from ex-holotype CGMCC 3.20476). (A, B) Upper and reverse views of culture on PDA 14 days after inoculation; (C to M) conidiophores, conidia, and intercalary conidia. Scale bars (C to M), 10 μm.

Pseudogymnoascus zhejiangensis Zhang, Han, and Liang, sp. nov. (Fig. 7). MycoBank number: MB 840439. Etymology: refers to the region from which the fungus was isolated. Diagnosis: similar to *P. lindneri*, *P. turneri*, and *P. yunnanensis* but differs in the obovoid, subglobose intercalary conidia. Type: China, Zhejiang Province, Ningbo City, Moon Lake, 29.871117N, 121.544218E, isolated from green belt soil, 16 August 2019, Z.Y. Zhang. (Holotype HMAS 350321, stored in a metabolically inactive state; ex-holotype culture CGMCC 3.20476 = GZUIFR 21.810, *ibid*., GZUIFR 21.811; *ibid*., GZUIFR 21.812.) GenBank: MZ444075, MZ444076, MZ444077 (ITS); MZ444102, MZ444103, MZ444104 (LSU); MZ490757, MZ490758, MZ490759 (*MCM7*); MZ488540, MZ488541, MZ488542 (*RPB2*); MZ488517, MZ488518, MZ488519 (*TEF*).

Description. Sexual morph: not observed. Asexual morph: colonies on PDA attaining 20 mm diameter after 14 days at 25°C, gradually increased from the edge to the center, velvety, floccose, margin entire, white, absent pigment and exudates; reverse pink, white at the edge. No growth at 37°C. Hyphae hyaline, branched, septate, smooth, 1 to 3 μm wide. Racquet hyphae absent. Conidiophores abundant, frequent branches, at acute angles, often 1 to 4 verticillate with 1 to 4 branches per whorl, secondary and tertiary branches can still branch again. Conidia abundant, normally borne terminally on verticillate branches or borne laterally and solitary on short protrusions or short side branches; subhyaline to hyaline, smooth-walled or rough; obovoid to globose, 2.5 to 4.5 by 2.5 to 4.0 μm (*n* = 50); clavate, long obovoid, 5 to 9 by 2.5 to 4 μm (*n* = 50). Intercalary conidia are borne on the verticillate hyphae or hyphae, solitary, smooth-walled or rough, obovoid, subglobose to globose, 3.5 to 4.5 by 3.0 to 4.0 μm (*n* = 50).

Substrate: Soil. Distribution: Ningbo City, Zhejiang Province, China.

Notes. Morphologically, *Pseudogymnoascus zhejiangensis* resembles *P. lindneri*, *P. turneri*, and *P. yunnanensis* because of the obovoid, globose conidia. However, *P. zhejiangensis* differs from *P. lindneri*, *P. turneri*, and *P. yunnanensis* in that it has obovoid, subglobose intercalary conidia (the intercalary conidia of *P. linderi* and *P. turneri* are globose to truncate, and those of *P. yunnanensis* are reniform, fusiform, and truncated at both ends) (27). Phylogenetically, three isolates of *P. zhejiangensis* formed one clade and share a sister relationship to three undescribed isolates (12NJ13, 17WV06, and 22984-1-I1) with high BS (Fig. 1). However, we did not compare morphological characteristics between *P. zhejiangensis* and another three isolates within *Pseudogymnoascus* because of the lack of morphological description of these three isolates (24).

**FIG 8:**
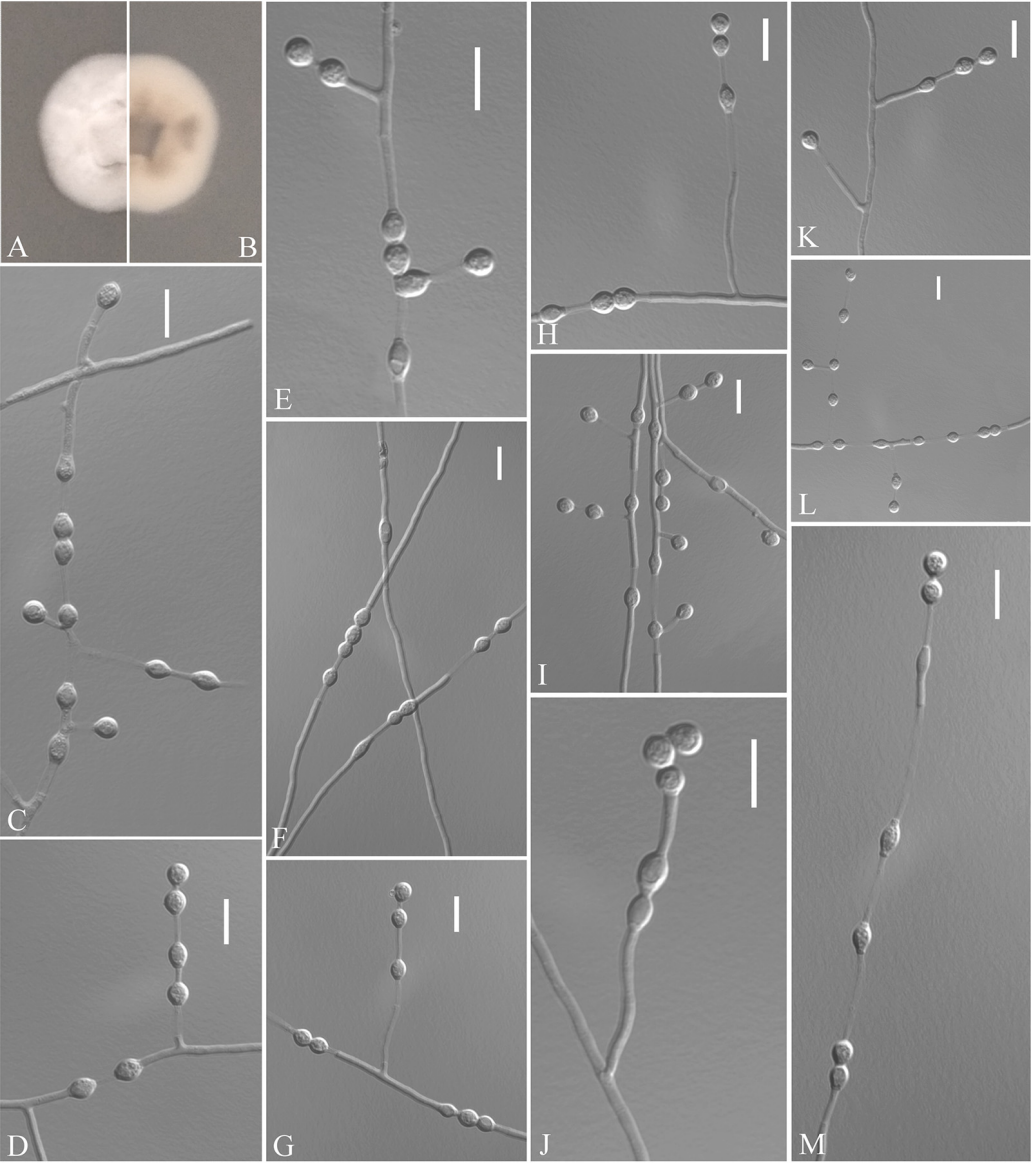
*Solomyces guizhouensis* (from ex-holotype CGMCC 3.20477). (A, B) Upper and reverse views of culture on PDA 14 days after inoculation; (C to M) terminal, lateral conidia, and intercalary conidia. Scale bars (C to M), 10 μm.

Solomyces guizhouensis Zhang, Han, and Liang, sp. nov. (Fig. 8). MycoBank number: MB 840440. Etymology: refers to Guizhou, the province where the isolate was collected. Diagnosis: *Solomyces guizhouensis* differs from other species by the presence of 2 to 3 conidia in chains and 2 to 3 intercalary conidia in chains. Type: China, Guizhou Province, Anshun City, Anshun University, 26.244748N, 105.898997E, isolated from green belt soil, 5 September 2019, Z.Y. Zhang. (Holotype HMAS 350319, stored in a metabolically inactive state; ex-holotype culture CGMCC 3.20477 = GZUIFR 21.804.) GenBank: MZ444069 (ITS); MZ444096 (LSU); MZ490751 (*MCM7*); MZ488534 (*RPB2*); MZ488511 (*TEF*).

Description. Sexual morph: not observed. Asexual morph: colonies on PDA, reaching 16 to 17 mm diameter after 14 days at 25°C, floccose, margins regular, white, absent pigment and exudates; reverse white. No growth at 37°C. Hyphae abundant, smooth and thin-walled, septate, 1.5 to 3.0 μm wide. Conidia terminal and laterally borne on hyphae, short protrusions, or side branches; solitary, sometimes 2 to 3 in chains, hyaline, smooth or rough walled, obovoid, subglobose to globose, pyriform, 4.0 to 7.0 by 4.0 to 6.0 μm (*n* = 50). Intercalary conidia abundant, solitary or 2 to 3 in chains, hyaline, smooth or rough walled, olivary, subglobose to globose, 4.5 to 8.5 by 3.5 to 5.0 μm (*n* = 50). Lateral branches may emerge from intercalary conidia.

Substrate: soil. Distribution: Anshun City, Guizhou Province, China. Material examined: China, Guizhou Province, Anshun City, People’s Hospital of Anshun City Guizhou Province, 26.247091N, 105.967968E, isolated from green belt soil, 5 September 2019, Z.Y. Zhang, GZUIFR 21.805, *ibid*., GZUIFR 21.806. GenBank: MZ444070, MZ444071 (ITS); MZ444097, MZ444098 (LSU); MZ490752, MZ490753 (*MCM7*); MZ488535, MZ488536 (*RPB2*); MZ488512, MZ488513 (*TEF*).

Notes. Morphologically, *Solomyces guizhouensis* is distinguished from other species of *Solomyces* by the presence of 2 to 3 conidia in chains and 2 to 3 intercalary conidia in chains. *Solomyces guizhouensis* is phylogenetically allied to Solomyces ramosus (Fig. 1), but they can be easily distinguished (see notes on *S. ramosus* [22]).

**FIG 9:**
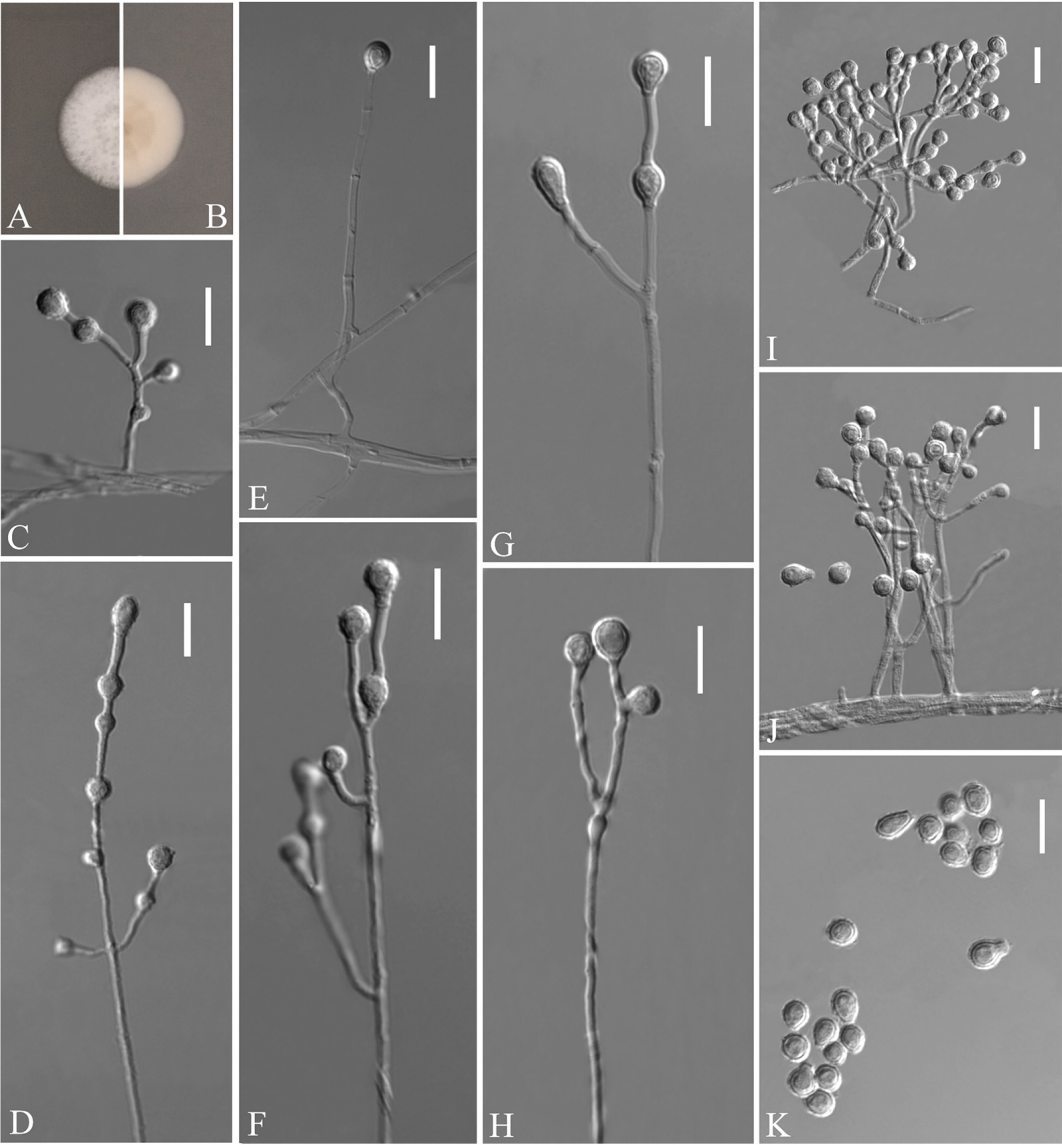
*Solomyces ramosus* (from ex-holotype CGMCC 3.20478). (A, B) Upper and reverse views of culture on PDA 14 days after inoculation; (C to H) terminal, lateral conidia, and intercalary conidia; (I and J) ramose of conidiophore; (K) conidia. Scale bars (C to K), 10 μm.

Solomyces ramosus Zhang, Han, and Liang, sp. nov. (Fig. 9). MycoBank number: MB 840442. Etymology: referring to the ramose of its conidiophore. Diagnosis: *Solomyces ramosus* differ from other species by the presence of ramose conidiophores. Type: China, Shanghai City, Ruijin Hospital, Shanghai Jiao Tong University School of Medicine, 31.212090N, 121.467721E, isolated from green belt soil, 15 August 2019, Z.Y. Zhang. (Holotype HMAS 350323, stored in a metabolically inactive state; ex-holotype culture CGMCC 3.20478 = GZUIFR 21.817, *ibid*., GZUIFR 21.818.) GenBank: MZ444082, MZ444083 (ITS); MZ444109, MZ444110 (LSU); MZ490764, MZ490765 (*MCM7*); MZ488547, MZ488548 (*RPB2*); MZ488524, MZ488525 (*TEF*).

Description. Sexual morph: not observed. Asexual morph: colonies on PDA reaching 17 mm diameter after 14 days at 25°C, slightly felty to floccose, margin identified, white; reverse white; absent pigment and exudates. No growth at 37°C. Hyphae abundant, smooth, hyaline, branched, septate, 1.0 to 3.5 μm wide. Conidiophores abundant, branches, at acute angles, 1 to 2 verticillate with 1 to 4 branches per whorl. Conidia terminal and laterally borne on hyphae, short protrusions, or side branches, solitary, hyaline, obovoid, subglobose, smooth or rough walled, 5 to 8.5 by 4.0 to 5.5 μm (*n* = 50). Intercalary conidia abundant, globose, olivary, subglobose to globose, 3.5 to 6.5 by 3.5 to 5.0 μm (*n* = 50).

Substrate: soil. Distribution: Shanghai City, China.

Notes. Morphologically, *Solomyces ramosus* is distinguished from other species of *Solomyces* by the presence of ramose conidiophores (22). Phylogenetically, our two new isolates of S. ramosus formed one clade and share a sister relationship to *S. guizhouensis* with high BS (Fig. 1), which indicates that they are distinct species.

*Zongqia* Zhang and Han, gen. nov. MycoBank number: MB 840447. Typification: Zongqia sinensis Zhang and Han. Etymology: in honor of Zong-Qi Liang, acknowledging his contributions to our group. Diagnosis: in addition to the phylogenetic distinctions (Fig. 1 to 2), *Zongqia* differs from *Pseudeurotium* by the presence of chains of conidia, conidiophores degenerated into conidiophore cells, clavate conidiophores cells.

Description. Saprobic on the soil. Sexual morph: not observed. Asexual morph: hyphae branched, septate, smooth. Conidiophores not observed and were degenerated into conidiophore cells. Conidiophores cells hyaline, cylindrical, clavate, occurring directly from the hyphae, smooth-walled, solitary. Conidia aseptate, smooth-walled, one-celled, solitary or chains, obovate, subglobose, fusiform, cylindrical, clavate. Chlamydospores not observed.

Notes. The new genus *Zongqia* is introduced here based on phylogeny and morphological evidence. Until now, the *Thelebolales* consisted of 23 genera (22, 28). In five-loci (ITS, LSU, *MCM7*, *RPB2*, and *EF1A*; Fig. 1) and two-loci (ITS and LSU; Fig. 2) phylogenetic analyses, *Zongqia* was related to *Pseudeurotium* with high support values (1 PP/100% BS). However, because no ITS, LSU, *MCM7*, *RPB2*, and *EF1A* sequence data were reported for *Ascophanus*, *Ascozonus*, *Caccobius*, *Coprobolus*, *Leptokalpion*, *Neelakesa*, and *Pseudascozonus* (22), we could not compare the phylogenetic relationships between these genera and *Zongqia*. Morphologically, because there is no record of the asexual stage of *Ascophanus*, *Ascozonus*, *Caccobius*, *Coprobolus*, *Leptokalpion*, *Neelakesa*, and *Pseudascozonus* in the literature (29), we could not compare the morphology between these genera and *Zongqia*. Of the remaining genera, *Zongqia* is similar to *Pseudeurotium*, but there are still noteworthy differences between them. *Zongqia* is distinguished from *Pseudeurotium* by the presence of chains of conidia, conidiophores degenerated into conidiophore cells, clavate conidiophores cells, and no observed sexual morph.

**FIG 10:**
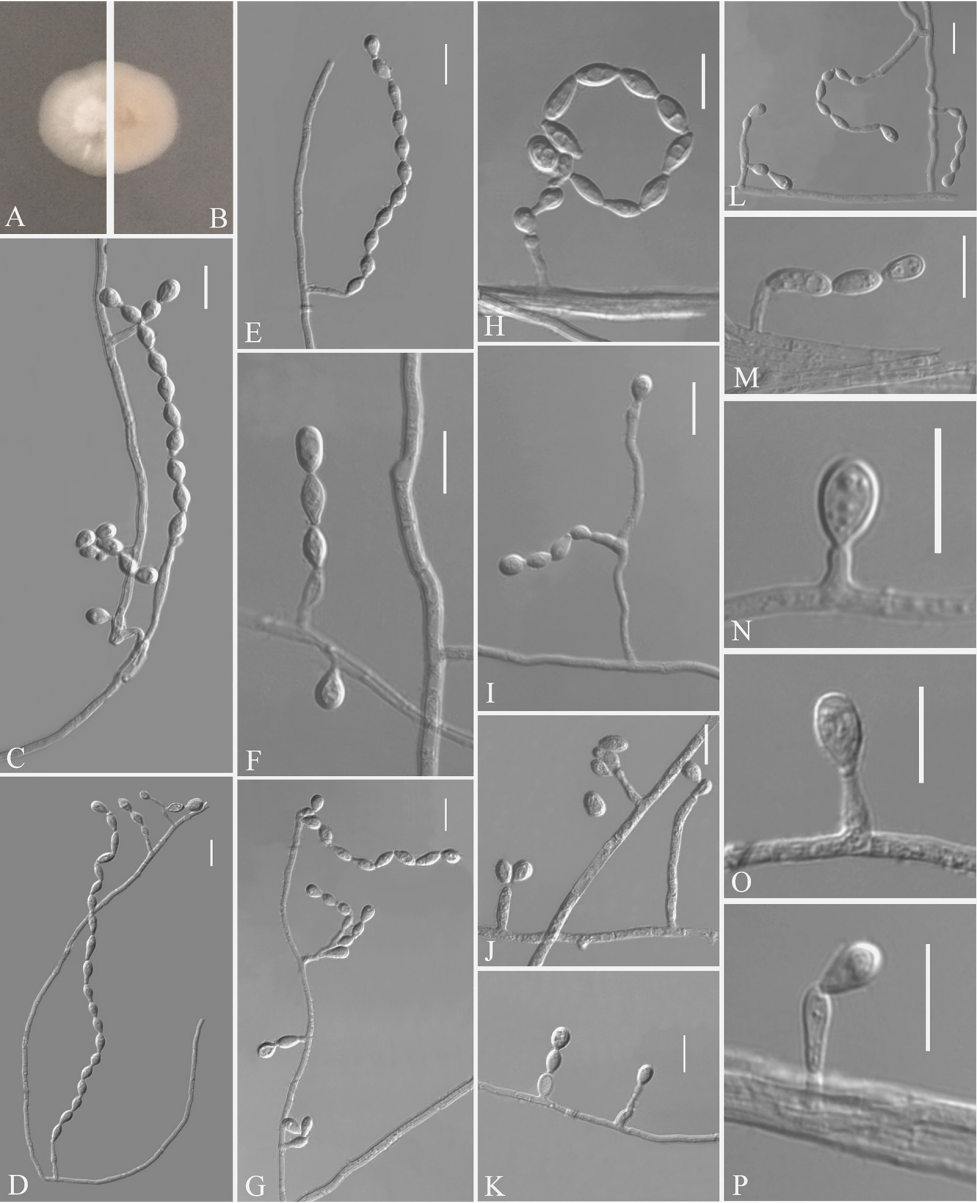
*Zongqia sinensis* (from ex-holotype CGMCC 3.20471). (A, B) Upper and reverse views of culture on PDA 14 days after inoculation; (C to E, H, I, L, M) conidia chains; (F) conidia borne on hyphae; (G) differentiation of conidiophore cells; (J) two conidia on the apex of conidiophore cells; (K) degenerated conidiophores; (N to P) solitary conidia. Scale bars (C to P), 10 μm.

Zongqia sinensis Zhang and Han, sp. nov. (Fig. 10). MycoBank number: MB 840448. Etymology: named after China where the species is distributed. Diagnosis: the main diagnostic criteria of the species *Zongqia sinensis* are presence of chains of conidia, conidiophores degenerated into conidiophore cells, clavate conidiophores cells. Type: China, Guizhou Province, Guiyang, The Aﬃliated Hospital of Guizhou Medical University, 26.594218N, 106.713166E, isolated from green belt soil, 13 September 2019, Z.Y. Zhang. (Holotype HMAS 350325, stored in a metabolically inactive state; ex-holotype culture CGMCC 3.20471 = GZUIFR 21.823, *ibid*., GZUIFR 21.824.) GenBank: MZ444088, MZ444089 (ITS); MZ444115, MZ444116 (LSU); MZ490770, MZ490771 (*MCM7*); MZ488553, MZ488554 (*RPB2*).

Description. Sexual morph: not observed. Asexual morph: colonies grow slowly on PDA, reaching 11 to 13 mm diameter after 14 days at 25°C, suborbicular, white, floccose, margins regular; reverse white, no growth at 37°C. Hyphae hyaline, branched, septate, smooth, 1.5 to 3.5 μm wide. Conidiophores not observed but degenerated into conidiophore cells. Conidiophore cells hyaline, cylindrical, clavate, arising directly from the aerial hyphae, smooth-walled, solitary. Conidia aseptate, smooth-walled, one-celled, solitary, obovate to subobovoid, 5 to 9 by 3 to 5 μm (*n* = 50); or 2 to 20 in chains, obovate, subglobose, fusiform and obtuse at apex and base, sometimes cylindrical, clavate, 3.5 to 8.5 (to 12) by 2.5 to 4.5 μm (*n* = 50). Chlamydospores not observed.

Substrate: soil. Distribution: Guiyang City, Guizhou Province, China. Material examined: China, Guizhou Province, Guiyang, Guizhou University, 26.444504N, 106.669296E, isolated from green belt soil, 13 September 2019, Z.Y. Zhang, GZUIFR 21.825. GenBank: MZ444090 (ITS); MZ444117 (LSU); MZ490772 (*MCM7*); MZ488555 (*RPB2*). Guizhou Province, Guiyang, Qianlingshan Park, 26.592019N, 106.695434E, isolated from green belt soil, 13 September 2019, Z.Y. Zhang, GZUIFR 21.826. GenBank: MZ444091 (ITS); MZ444118 (LSU); MZ490773 (*MCM7*); MZ488556 (*RPB2*).

Notes. Based on multilocus phylogenetic analyses (Fig. 1 and 2) and similar morphological characteristics, the four strains are regarded as the same species, which cluster together very well and form a single clade separated from other species of *Thelebolales*. Morphologically, Zongqia sinensis is the only species that produces the conidia chains in this order. Therefore, based on both morphological and phylogenetic evidence, Z. sinensis is proposed as a novel species as a type of *Zongqia*.

**FIG 11:**
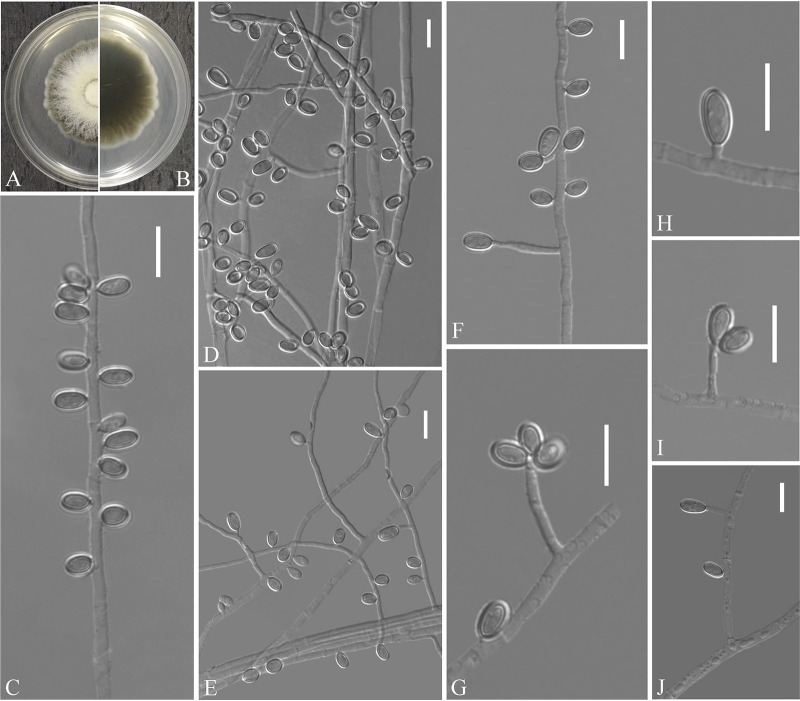
*Scedosporium haikouense* (from ex-holotype CGMCC 3.20468). (A, B) Upper and reverse views of culture on PDA 14 days after inoculation; (C to J) conidiogenous cells and conidia. Scale bars (C to J), 10 μm.

Scedosporium haikouense Zhang, Han, and Liang, sp. nov. (Fig. 11). MycoBank number: MB 840443. Etymology: refers to Haikou, the city where the isolate was collected. Diagnosis: the main diagnostic criteria of the species *Scedosporium haikouense* are abundant ovoid, ellipsoidal, subcylindrical conidia, conidiogenous cells solitary or 2 to 3 fascicled conidia, and absent pigment and exudates and lack of synnemata. Type: China, Hainan Province, Haikou City, Hainan university Haidian Campus, 20.059602N, 110.330436E, isolated from green belt soil, 28 August 2019, Z.Y. Zhang. (Holotype HMAS 350313, stored in a metabolically inactive state; ex-holotype culture CGMCC 3.20468 = GZUIFR 21.833, *ibid*., GZUIFR 21.834.) GenBank: MZ469289, MZ469290 (ITS); MZ488563, MZ488564 (*BT2*).

Description. Sexual morph: not observed. Asexual morph: colonies on PDA attaining 54 to 56 mm diameter after 5 days at 25°C, fluffy, flavescens to white, gray at margins, annular at the center, margin slightly undulate; reverse cream-yellow to black; absent pigment and exudates. Colonies on PDA attaining 68 to 70 mm diameter after 5 days at 37°C. Hyphae hyaline, branched, septate, smooth-walled, 0.5 to 5.5 μm wide. Conidiophores solitary, usually reduced to conidiogenous cells, arising terminally or laterally from hypha, hyaline, smooth-walled, cylindrical, 1.5 to 26.0 by 1.0 to 2.0 μm (*n* = 50). Conidia are borne on hyphae, short protrusions, or side branches, one-celled, solitary, or 2 to 3 fascicled, pale brown to brown, ovoid, ellipsoidal, subcylindrical and bilaterally compressed, rounded at the ends, 5.0 to 9.0 by 3.0 to 4.5 μm (*n* = 50). Synnemata not observed.

Substrate: soil. Distribution: Haikou City, Hainan Province, China.

Notes. Phylogenetically, *Scedosporium haikouense* is closely related to Scedosporium rarisporum, Scedosporium cereisporum, and S. aurantiacum. However, *S. haikouense* can be distinguished from S. rarisporum by the presence of abundant ovoid, ellipsoidal, subcylindrical conidia (30), from *S. cereisporum* by the solitary conidiogenous cells, solitary or 2 to 3 fascicled conidia (31), and from S. aurantiacum by the absent pigment and exudates and lack of synnemata (32).

**FIG 12:**
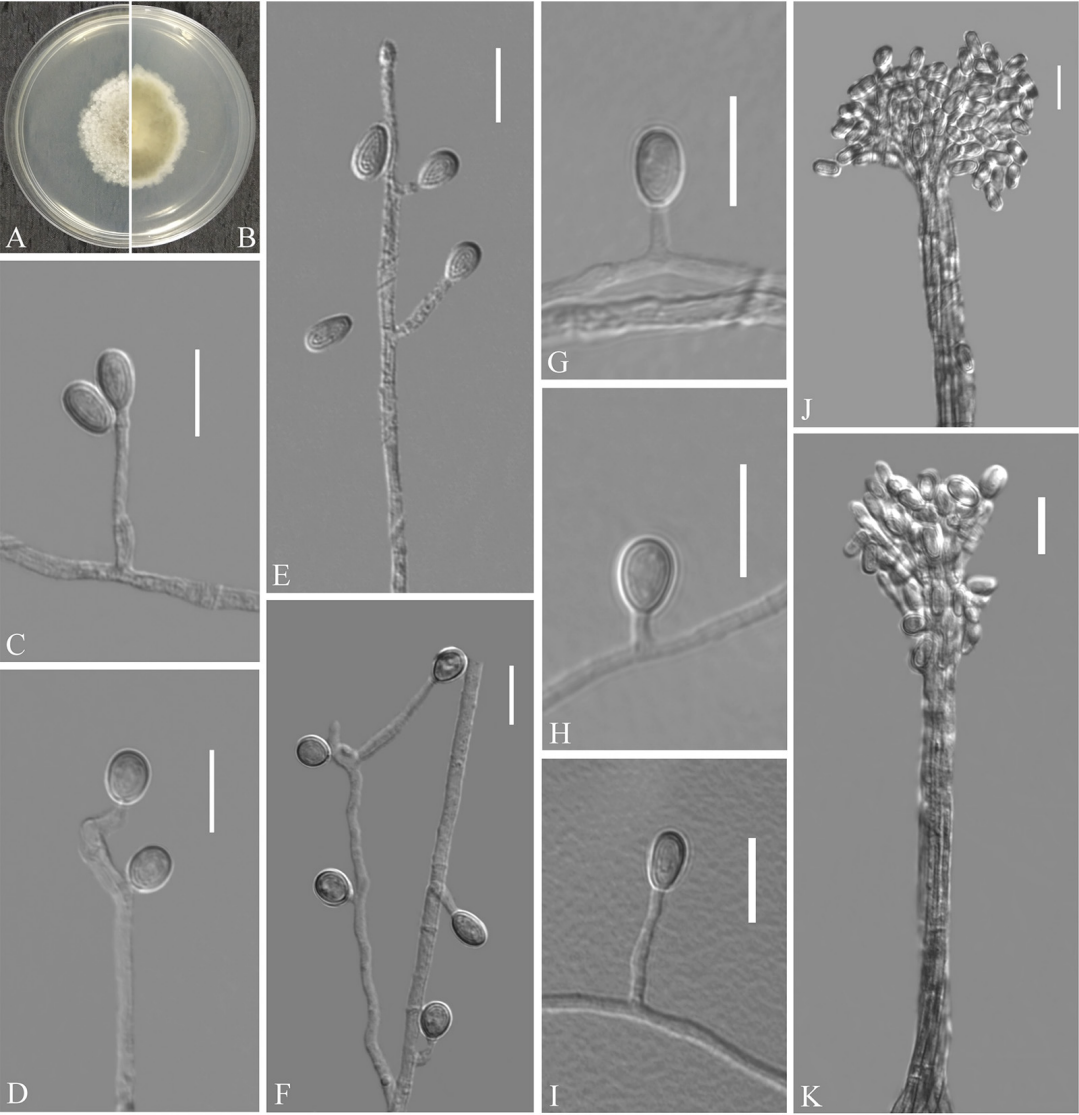
*Scedosporium hainanense* (from ex-holotype CGMCC 3.20469). (A, B) Upper and reverse views of culture on PDA 14 days after inoculation; (C to I) conidiogenous cells and conidia; (J and K) synnematous conidiomata. Scale bars (C to J), 10 μm.

Scedosporium hainanense Zhang, Han, and Liang, sp. nov. (Fig. 12). MycoBank number: MB 840445. Etymology: refers to Hainan, the province where the isolate was collected. Diagnosis: similar to S. apiospermum but differs in the ellipsoidal conidia. Type: China, Hainan Province, Sanya City, Hainan Tropical Ocean University, 18.311670N, 109.534152E, isolated from green belt soil, 26 August 2019, Z.Y. Zhang. (Holotype HMAS 350311, stored in a metabolically inactive state; ex-holotype culture CGMCC 3.20469 = GZUIFR 21.829.) GenBank: MZ469285 (ITS); MZ488559 (*BT2*).

Description. Sexual morph: not observed. Asexual morph: colonies on PDA attaining 38 to 43 mm diameter after 5 days at 25°C, cottony, floccose, light gray, margins irregular; reverse peltricolor, white to margins; absent pigment and exudates. Colonies on PDA attaining 64 to 66 mm diameter after 5 days at 37°C. Hyphae hyaline, branched, septate, smooth-walled, 0.5 to 4.5 μm wide. Conidiophores solitary, often consisting of a single conidiogenous cell, or arranged in whorls of 2 to 3 conidiogenous cells, arising terminally or laterally from hypha, undifferentiated hypha, short-stalked, or inside branches. Conidiogenous cells annellidic, hyaline, thin- and smooth-walled, lateral or terminal, cylindrical or slightly broad at the base, sometimes with several annellations at the top with the age, 2.5 to 33.0 by 1.0 to 2.5 μm (*n* = 50). Conidia are borne on hyphae, short protrusions, or side branches, one-celled, solitary, hyaline, ovoid, 5.0 to 8.0 by 2.5 to 6.0 μm (*n* = 50), ellipsoidal, 5.5 to 7.0 by 5.0 to 5.5 μm (*n* = 50). Conidiomata synnematous, erect, consisting of a cylindrical stipe, hyaline, smooth-walled; conidia cylindrical or claviform with a truncated base, 4.5 to 8.5 by 2.5 to 3.5 μm (*n* = 50).

Substrate: soil. Distribution: Sanya and Danzhou City, Hainan Province, China. Material examined: China, Hainan Province, Sanya City, Seaside parks, 18.272349N, 109.479274E, isolated from green belt soil, 26 August 2019, Z.Y. Zhang, GZUIFR 21.828. GenBank: MZ469284 (ITS); MZ488558 (*BT2*). Hainan Province, Danzhou City, Hainan University Danzhou Campus, 19.508080N, 109.494579E, isolated from green belt soil, 27 August 2019, Z.Y. Zhang, GZUIFR 21.827. GenBank: MZ469283 (ITS); MZ488557 (*BT2*).

Notes. Morphological and phylogenetic data (Fig. 3) support our strains as new species of *Scedosporium hainanense*. *Scedosporium hainanense* is phylogenetically closely related to S. apiospermum complex that comprises Scedosporium angustum, S. apiospermum, S. boydii, S. ellipsoideum, and Scedosporium fusarium. However, *S. hainanense* can be distinguished from S. apiospermum by the ellipsoidal conidia. We did not compare morphological characteristics between S. hainanense and the S. apiospermum complex (S. angustum, S. apiospermum, S. boydii, *S. ellipsoideum*, and Scedosporium fusarium) because of the lack of asexual morph descriptions of these species (33).

**FIG 13:**
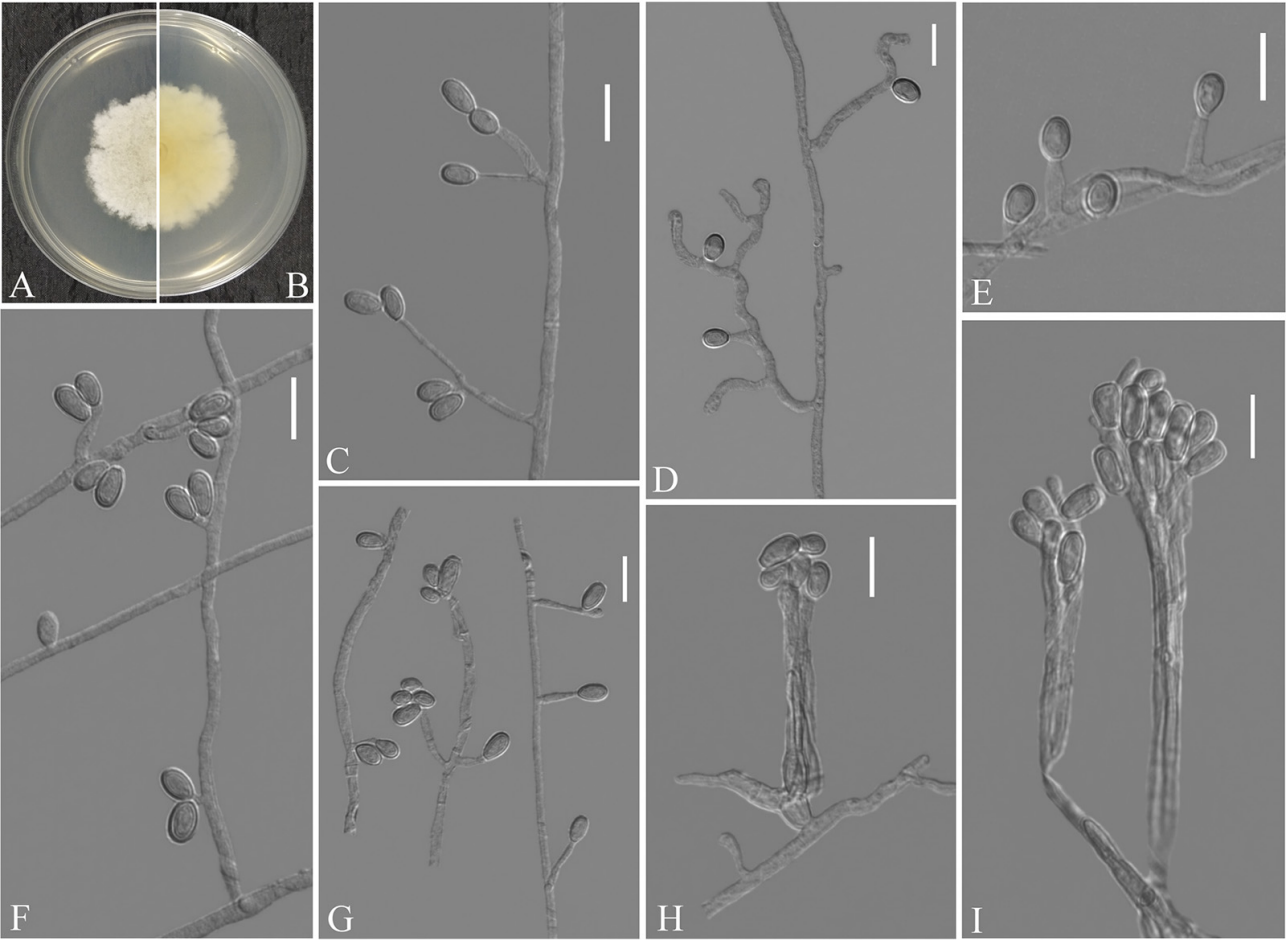
*Scedosporium multisporum* (from ex-holotype CGMCC 3.20470). (A, B) Upper and reverse views of culture on PDA 14 days after inoculation; (C to G) conidiogenous cells and conidia; (H and I) synnematous conidiomata. Scale bars (C to I), 10 μm.

Scedosporium multisporum Zhang, Han, and Liang, sp. nov. (Fig. 13). MycoBank number: MB 840446. Etymology: referring to the 2 to 3 fascicled conidia. Diagnosis: similar to S. apiospermum complex but differs in the presence of 2 to 3 fascicled conidia, conidiomata synnematous. Type: China, Hunan Province, Huaihua City, Huaihua University, 27.572703N, 110.023832E, isolated from green belt soil, 12 August 2019, Z.Y. Zhang. (Holotype HMAS 350312, stored in a metabolically inactive state; ex-holotype culture CGMCC 3.20470 = GZUIFR 21.830, *ibid*., GZUIFR 21.831; *ibid*., GZUIFR 21.832.) GenBank: MZ469286, MZ469287, MZ469288 (ITS); MZ488560, MZ488561, MZ488562 (*BT2*).

Description. Sexual morph: not observed. Asexual morph: colonies on PDA attaining 45 to 50 mm diameter after 5 days at 25°C, cottony, powdery at the center; reverse white, light yellow at the center; absent pigment and exudates. Colonies on PDA attaining 70 to 73 mm diameter after 5 days at 37°C. Hyphae hyaline, branched, septate, smooth-walled, 1.0 to 4.0 μm wide. Conidiophores solitary, often consisting of a single conidiogenous cell, or arranged in whorls of 2 to 3 conidiogenous cells, arising terminally or laterally from hypha, undifferentiated hypha, short-stalked, or inside branches. Conidiogenous cells annellidic, hyaline, thin- and smooth-walled, lateral or terminal, cylindrical or slightly broad at the base, sometimes with several annellations at the top with the age, 0.5 to 16.0 by 1.0 to 3.5 μm (*n* = 50). Conidia are borne on hyphae, short protrusions, or side branches, one-celled, solitary, or 2 to 3 fascicled, hyaline, ovoid to subglobose, 3.0 to 7.5 by 3.0 to 5.0 μm (*n* = 50). Conidiomata synnematous, erect, consisting of a cylindrical stipe, hyaline, smooth-walled; conidia cylindrical, ovoid, long ovoid with a truncated base, 5.0 to 10.0 by 2.0 to 4.0 μm (*n* = 50).

Substrate: soil. Distribution: Huaihua City, Hunan Province, China.

Notes. *Scedosporium multisporum* is phylogenetically closely related to the S. apiospermum complex that comprises *S. angustum*, S. apiospermum, S. boydii, *S. ellipsoideum*, and S. fusarium. However, S. multisporum is distinguished from other species of *Scedosporium* by the presence of 2 to 3 fascicled conidia, conidiomata synnematous (33).

Parascedosporium sanyaense (Han, Zheng, Luo, Wang, and Liang 2017) Zhang, Han, and Liang 2021, comb. nov. MycoBank: MB 818105. Basionym: Scedosporium sanyaense (see reference 30).

Description: Y.F. Han, Huan Zheng, Y. Luo, Y.R. Wang, and Z.Q. Liang 2017.

Notes. In 2017, Han et al. introduced S. sanyaense to the genus *Scedosporium*, based on morphological and internal transcribed spacers (ITS) phylogenetic analysis (30). However, in our phylogenetic study, *S. sanyaense* is placed in the genus *Parascedosporium*. Therefore, we propose a new combination for that species.

## DISCUSSION

The hair baiting technique was first used to isolate keratinophilic fungi from the soil by Vanbreuseghem (34) and has become applied widely. So far, the investigation of such resources is still dominated by traditional isolated cultures and baiting with materials of human or animal origin, such as feathers (35), horsehair (4), wool (36), human hair (37), and human nails (38). Only a small number of studies have used next-generation sequencing technologies (39).

Taxonomy and phylogenetic identification of fungi remain significant challenges (40). One of the main fundamental needs in fungal ecology is a strong taxonomic basis, which is dependent on advances in nucleic acid sequence technology. However, some researchers have relied too much on these techniques to the complete exclusion of fungal isolation and characterization using classical methods. While bacterial microbiome studies have relatively reliable taxonomic identification using 16S ribosomal DNA (rDNA) and even metagenome sequencing, mycobiome studies are still few and far between, with limited taxonomic interpretation capabilities. Indeed, phenotypic and culture-based studies remain an invaluable tool for fungal biology and ecology (41). The advantage of placing these organisms in pure culture is, of course, that almost all aspects of their biology can be studied, which may help to understand how they function in their natural ecological context. Thus, many challenges remain in studying the hundreds of niches on Earth that may be inhabited by fungi, not only to demonstrate their presence in these niches but also to culture them in pure form and store them properly for further study (42).

The ability of microorganisms to degrade recalcitrant materials has been widely explored for environmental remediation and industrial production. Significant success has been achieved with single strains, but the focus is now on the use of microbial consortia because of their functional stability and efficiency (43). The keratin degradation process requires the synergistic action of different enzymes, such as endoproteases, exoproteases, oligopeptidases, and disulfide reductases (44); thus, this process involves the synergistic cooperation of multiple species. We did not isolate purified fungal strains directly from feathers after enrichment using hair bating but did isolate members of the fungal community from the soil. Therefore, we could not determine whether the obtained strains are keratinophilic fungi and whether they are able to degrade and utilize keratin. However, numerous studies have shown that many members of *Thelebolales* and *Scedosporium* are indeed keratinophilic fungi (45–48). Hence, we think that our obtained strains are the keratinophilic fungi and should at least be constituent members of the keratin-degrading fungal consortia, although it is not clear what role they play in this consortium. In this study, 10 new species were identified and introduced, not only contributing to the further understanding of the keratin-degrading fungal community but also accumulating strains for future artificially constructed keratin-degrading microbial consortia.

## MATERIALS AND METHODS

### Sampling, fungal isolation, and morphology.

Soil samples were collected from Guizhou, Hunan, Zhejiang, Yunnan, Fujian, Hainan, Jiangxi, Guangdong, and Zhejiang provinces in southern China and transported to the laboratory in Ziploc plastic bags. The soil samples were processed using the method we described previously (22). Briefly, clean and sterile chicken feathers were placed in a sterile petri dish after the soil sample was added, wetted with distilled water, and incubated at room temperature for 1 month. Fungi were isolated using a conventional dilution technique based on Sabouraud’s dextrose agar (SDA; 10 g of peptone, 40 g of dextrose, 20 g of agar, 1 liter of ddH_2_O) supplemented with chloramphenicol and cycloheximide, and the purification of the strains was performed using potato dextrose agar (PDA; Shanghai Bio-way Technology Co., Ltd., China) (20, 22). Colonies on PDA were incubated after 14 days at 25°C, and the cultures were placed to slowly dry at 50°C to produce the holotype. Holotypes were deposited in the Mycological Herbarium of the Institute of Microbiology, Chinese Academy of Sciences, Beijing, China (HMAS). All strains were deposited in the Institute of Fungus Resources, Guizhou University (GZUIFR, the Herbarium of Guizhou Agricultural College, code GZAC), and the ex-type strains were also deposited in the China General Microbiological Culture Collection Center (CGMCC). The living cultures were stored in a metabolically inactive state, i.e., kept in sterile 30% glycerol in a −80°C freezer. Macroscopic and morphological characterization of the colonies was performed on PDA incubated for 14 days in the dark at 25°C. The characterization and measurement of fungal microscopic characteristics were performed in 25% lactic acid. Images were obtained using an optical microscope (OM; DM4 B, Leica, Germany) with differential interference contrast (DIC). Taxonomic descriptions and nomenclature were deposited at MycoBank (https://www.mycobank.org/).

### DNA extraction, PCR amplification, and sequencing.

Total genomic DNA was extracted from fungal mycelia using the BioTeke fungus genomic DNA extraction kit (DP2032, BioTeke, Beijing, China) following the manufacturer’s instructions. Multiple loci were amplified and sequenced for each new isolate, and the primer sets are listed in Table 2. Amplification conditions were carried out as in the original literature where the primers were reported. The PCR thermal cycle programs for each locus amplification were performed as in the original literature where the primers were reported. The PCR products were sequenced with the amplified primers at a commercial sequencing service provider (Shanghai Sangon Biological Engineering Technology & Services Co., Shanghai, China) in an ABI 3730xl DNA analyzer using the Sanger method. The consensus sequences were obtained using the SeqMan software v. 7 (DNASTAR Lasergene, Madison, WI, USA).

**TABLE 2:** Primers used in this study

Locus	Primer	Primer sequence 5′ to 3′	Orientation	Reference
ITS	ITS1	TCCGTAGGTGAACCTGCGG	Forward	56
	ITS4	TCCTCCGCTTATTGATATGC	Reverse	56
Beta-tubulin (*BT2*)	Bt2a	GGTAACCAAATCGGTGCTGCTTTC	Forward	57
	Bt2b	ACCCTCAGTGTAGTGACCCTTGGC	Reverse	57
Large subunit ribosomal DNA (LSU)	LROR	ACCCGCTGAACTTAAGC	Forward	58
	LR7	TACTACCACCAAGATCT	Reverse	59
Translation elongation factor 1-alpha (*TEF1-α*)	983F	GCYCCYGGHCAYCGTGAYTTYAT	Forward	60
	EF1-2218R	ATGACACCRACRGCRACRGTYTG	Reverse	60
RNA polymerase II subunit 2 (*RPB2*)	fRPB2-7cF	ATGGG[T/C]AA[A/G]CAAGC[T/C]ATGGG	Forward	61
	RPB2-3053bR	TGRATYTTRTCRTCSACCAT	Reverse	62
Minichromosomal maintenance protein 7 (*MCM7*)	MCM7-709	ACIMGIGTITCVGAYGTHAARCC	Forward	63
	MCM7-1348	GAYTTDGCIACICCIGGRTCWCCCAT	Reverse	63

### Phylogenetic analysis.

The data sets were assembled based on the closest matches from the BLASTn search results and recently published data. Sequences generated from each locus were analyzed along with other sequences retrieved from GenBank. The individual loci matrix was aligned with MAFFT v7.037b (49) and was further edited manually, where necessary, using BioEdit v.7.0.9.0 (50). The best-fit model of maximum likelihood (ML) and Bayesian analyses of each locus were estimated using IQ-TREE’s ModelFinder function (51) using the Akaike Information Criterion (AIC).

Phylogenetic analyses of the combined aligned data were performed under ML and Bayesian inference (BI). ML analyses were performed with IQ-TREE v. 1.6.11 (52). Bootstrap analyses were performed using the ultrafast bootstrap approximation (53) with 10,000 replicates, and bootstrap support (BS) greater than 70% was considered significantly supported. The BI was conducted with MrBayes v. 3.2.6 (54). Four Markov chains were run for two runs from random starting trees for 5 million generations, and trees were sampled every 1,000 generations. The first 25% of the sampled trees were discarded as burn-in, and the remaining ones were used to reconstruct a majority rule consensus tree and calculate Bayesian posterior probabilities (BPP) of the clades. The above analyses were carried out in PhyloSuite v1.16 (55).

### Data availability.

The sequences generated in this study can be found in GenBank. The accession numbers of the sequences deposited in GenBank are listed in Table 3.

**TABLE 3:** List of GenBank accession numbers of sequences used in this study^*a*^

Species	Strain	GenBank accession no.					
		ITS	LSU	*MCM7*	*RPB2*	*TEF1*	*BT2*
*Thelebolales*							
* * Alatospora acuminata	CBS 104.88	MH862121	MH873811				
* * Alatospora constricta	CCM F-11302	KC834040	KC834017				
* * Alatospora pulchella	CCM F-502	KC834039	KC834019				
* * Antarctomyces pellizaniae	UFMGCB 12416	KX576510					
* * Antarctomyces psychrotrophicus	CBS 100573	MH874317					
* * Cleistothelebolus nipigonensis	CBS 778.70	MH859938	MH871738				
* * Crinula caliciiformis	AFTOL-ID 272	KT225524	AY544680				
* * Epiglia gloeocapsae	CBS 126301	MH863968	MH875423				
	CBS 126302	MH863969	MH875424				
* * Geomyces auratus	CBS 108.14	KF039895	KF017864	KF017690	KF017746	KF017805	
* * Geomyces obovatus	CGMCC 3.18491	MT509362	MT509376	MT534202	MT534216	MT534227	
	CGMCC 3.18492	MT509363	MT509377	MT534203	MT534217	MT534228	
* * Gorgomyces honrubiae	CCM F-12003	KC834057	KC834028				
	CCM F-12696	KC834058					
* * Gymnostellatospora alpina	CBS 620.81	MH861383	MH873132				
* * Gymnostellatospora bhattii	CBS 760.71	MH860337	MH872092				
	CBS 761.71	MH860338	MH872093				
	CBS 762.71	MH860339	MH872094				
* * Holwaya mucida	NBRC 112552	LC425042	LC429385				
	TU 112863	MH752062	KX090844				
* * Leuconeurospora pulcherrima	CBS 343.76	KF049206	FJ176884		FJ238367	FJ238409	
* Leuconeurospora* sp.	02NH04	JX270349	KF017817	KF017648	KF017702	KF017757	
	15PA04	JX270479	KF017841	KF017669	KF017725	KF017781	
* * Miniancora allisoniens	CCM F-30487	KC834064					
* * Patinella hyalophaea	H.B.9739	KT876978	KT876978				
* * Pseudeurotium bakeri	CBS 128111	MH864831	MH876274				
	CBS 128112	MH864832	MH876275				
	CBS 128113	MH864833	MH876276				
	CBS 878.71	MH860393	MH872136				
* * Pseudeurotium hygrophilum	CBS 102670	AY129291	MH874401				
	CBS 102671	AY129292					
	S661	KP644137	KP644138				
* * Pseudeurotium ovale	CBS 389.54	MH857368	MH868913				
	CBS 454.62	MH858209	MH869809				
	CBS 531.71	MH860256	MH872019				
* *Pseudeurotium ovale var. *ovale*	UAMH 5825	KJ755521					
* * Pseudeurotium zonatum	CBS 126947	MH864346	MH875790				
	CBS 130172	MH865520	MH876956				
	CBS 329.36	AY129286	DQ470988		DQ470940	DQ471112	
	CBS 391.61	MH858096	MH869666				
* * Pseudogymnoascus appendiculatus	02NH11	JX270356	KF017819	KF017650	KF017704	KF017759	
	07MA02	JX270402	KF017827	KF017658	KF017712	KF017767	
* * Pseudogymnoascus catenatus	GZUIFR 21.813^*a*^	MZ444078	MZ444105	MZ490760	MZ488543	MZ488520	
	GZUIFR 21.814^*a*^	MZ444079	MZ444106	MZ490761	MZ488544	MZ488521	
	GZUIFR 21.815^*a*^	MZ444080	MZ444107	MZ490762	MZ488545	MZ488522	
	GZUIFR 21.816^*a*^	MZ444081	MZ444108	MZ490763	MZ488546	MZ488523	
* * Pseudogymnoascus destructans	20631.21	EU884921	KF017865	KF017691	KF017747	KF017806	
* * Pseudogymnoascus fujianensis	GZUIFR 21.819^*a*^	MZ444084	MZ444111	MZ490766	MZ488549	MZ488526	
	GZUIFR 21.820^*a*^	MZ444085	MZ444112	MZ490767	MZ488550	MZ488527	
	GZUIFR 21.821^*a*^	MZ444086	MZ444113	MZ490768	MZ488551	MZ488528	
	GZUIFR 21.822^*a*^	MZ444087	MZ444114	MZ490769	MZ488552	MZ488529	
* * Pseudogymnoascus guizhouensis	GZUIFR 376.1	MT509369	MT509383	MT534209	MT534223	MT534234	
	GZUIFR 376.2	MT509370	MT509384	MT534210	MT534224	MT534235	
	GZUIFR 376.3	MT509371	MT509385	MT534211	MT534225	MT534236	
* * Pseudogymnoascus lindneri	02NH05	JX270350	KF017818	KF017649	KF017703	KF017758	
	LHU.158	MN542212			MN541384	MN541383	
* * Pseudogymnoascus roseus	05NY06	JX270385	KF017824	KF017655	KF017709	KF017764	
	05NY08	JX270387	KF017825	KF017656	KF017710	KF017765	
	05NY09	JX270388	KF017826	KF017657	KF017711	KF017766	
* * Pseudogymnoascus shaanxiensis	GZUIFR 21.800^*a*^	MZ444065	MZ444092	MZ490747	MZ488530	MZ488507	
	GZUIFR 21.801^*a*^	MZ444066	MZ444093	MZ490748	MZ488531	MZ488508	
* * Pseudogymnoascus shaanxiensis	GZUIFR CY1.8	MT509367	MT509381	MT534207	MT534221	MT534232	
	GZUIFR HZ5.7	MT509366	MT509380	MT534206	MT534220	MT534231	
* * Pseudogymnoascus sinensis	CGMCC 3.18493	MT509364	MT509378	MT534204	MT534218	MT534229	
	CGMCC 3.18494	MT509365	MT509379	MT534205	MT534219	MT534230	
* Pseudogymnoascus* sp.	04NY11	JX270375	KF017821	KF017652	KF017706	KF017761	
	04NY17A	JX270378	KF017823	KF017654	KF017708	KF017763	
	10NY08	JX270432	KF017829	KF017659	KF017714	KF017769	
	10NY09	JX270433	KF017830	KF017660	KF017715	KF017770	
	10NY10	JX270434	KF017831		KF017716	KF017771	
	11MA03	JX270438	KF017832	KF017661	KF017717	KF017772	
	11MA05	JX270440	KF017833	KF017662	KF017718	KF017773	
	11MA07	JX270442	KF017834	KF017663	KF017719	KF017774	
	11MA08	JX270443	KF017835	KF017664	KF017720	KF017775	
	12NJ13	JX270459	KF017838	KF017667	KF017722	KF017778	
	15PA10B	KF039894	KF017842	KF017670	KF017726	KF017782	
	15PA11	JX270486	KF017843	KF017671	KF017727	KF017783	
	17WV03	JX270510	KF017844	KF017672	KF017728	KF017784	
	17WV06	JX270513		KF017673	KF017729	KF017785	
	18VA07	JX270527	KF017847	KF017675		KF017788	
	18VA08	JX270528	KF017848	KF017676	KF017731	KF017789	
	18VA12	JX270532	KF017849		KF017732	KF017790	
	18VA13	JX270533	KF017850		KF017733	KF017791	
	20KY08	JX270562	KF017851	KF017677	KF017734	KF017792	
	20KY10	JX270563	KF017852	KF017678	KF017735	KF017793	
	20KY12	JX270565	KF017853	KF017679	KF017736	KF017794	
	21IN01	JX270568	KF017854	KF017680	KF017737	KF017795	
	21IN05	JX270572	KF017855	KF017681	KF017738	KF017796	
	21IN10	JX270577	KF017856	KF017682	KF017739	KF017797	
	22984-1-I1	JX415262	KF017866	KF017692		KF017807	
	23014-1-I6	JX512256	KF017867	KF017693	KF017748	KF017808	
	24MN04	JX270612	KF017859	KF017685	KF017741	KF017800	
	24MN06	JX270614	KF017860	KF017686	KF017742	KF017801	
	24MN14	JX270622	KF017862	KF017688	KF017744	KF017803	
	24MN18	JX270626	KF017863	KF017689	KF017745	KF017804	
	A07MA10	KF039893	KF017828		KF017713	KF017768	
	MN-Mycosel-7	KF039899	KF017872	KF017698	KF017753	KF017813	
	RMF 7792	KF039898	KF017871	KF017697	KF017752	KF017812	
* * Pseudogymnoascus turneri	LHU 121	MN542213			MN541380	MN541379	
	Ps5	MN542214			MN541382	MN541381	
* * Pseudogymnoascus verrucosus	01NH08	JX270343	KF017816	KF017647	KF017701	KF017756	
	04NY16	JX270377	KF017822	KF017653	KF017707	KF017762	
	24MN13	JX270621	KF017861	KF017687	KF017743	KF017802	
	GZUIFR 21.802^*a*^	MZ444067	MZ444094	MZ490749	MZ488532	MZ488509	
	GZUIFR 21.803^*a*^	MZ444068	MZ444095	MZ490750	MZ488533	MZ488510	
* * Pseudogymnoascus yunnanensis	GZUIFR 21.807^*a*^	MZ444072	MZ444099	MZ490754	MZ488537	MZ488514	
	GZUIFR 21.808^*a*^	MZ444073	MZ444100	MZ490755	MZ488538	MZ488515	
	GZUIFR 21.809^*a*^	MZ444074	MZ444101	MZ490756	MZ488539	MZ488516	
* * Pseudogymnoascus zhejiangensis	GZUIFR 21.810^*a*^	MZ444075	MZ444102	MZ490757	MZ488540	MZ488517	
	GZUIFR 21.811^*a*^	MZ444076	MZ444103	MZ490758	MZ488541	MZ488518	
	GZUIFR 21.812^*a*^	MZ444077	MZ444104	MZ490759	MZ488542	MZ488519	
* * Ramgea ozimecii	CNF 2/9997	KY368752	KY368753				
* * Solomyces guizhouensis	GZUIFR 21.804	MZ444069	MZ444096	MZ490751	MZ488534	MZ488511	
	GZUIFR 21.805	MZ444070	MZ444097	MZ490752	MZ488535	MZ488512	
	GZUIFR 21.806	MZ444071	MZ444098	MZ490753	MZ488536	MZ488513	
* * Solomyces ramosus	GZUIFR 21.817	MZ444082	MZ444109	MZ490764	MZ488547	MZ488524	
	GZUIFR 21.818	MZ444083	MZ444110	MZ490765	MZ488548	MZ488525	
* * Solomyces sinensis	CGMCC 3.18498	MT509373	MT509387	MT534213		MT534238	
	CGMCC 3.18499	MT509374	MT509388	MT534214		MT534239	
	CGMCC 3.18500	MT509375	MT509389	MT534215		MT534240	
* Solomyces* sp.	15PA02	JX270477	KF017840		KF017724	KF017780	
	17WV02	JX270509	KF017845		KF017730	KF017786	
* * Thelebolus balaustiformis	MUT 2357	NR_159056	NG_067559				
* * Thelebolus globosus	CBS 113940	MH862951	NG_067263				
* * Thelebolus spongiae	MUT 2359	MG813185	MG816493				
Undetermined	12NJ08	JX270454	KF017836	KF017665		KF017776	
	12NJ10	JX270456	KF017837	KF017666	KF017721	KF017777	
	17WV09	JX270515	KF017846	KF017674		KF017787	
	23WI08	JX270598	KF017858			KF017799	
	23WI14	JX270604		KF017684			
* * Zongqia sinensis	GZUIFR 21.823^*a*^	MZ444088	MZ444115	MZ490770	MZ488553		
	GZUIFR 21.824^*a*^	MZ444089	MZ444116	MZ490771	MZ488554		
	GZUIFR 21.825^*a*^	MZ444090	MZ444117	MZ490772	MZ488555		
	GZUIFR 21.826^*a*^	MZ444091	MZ444118	MZ490773	MZ488556		
*Scedosporium* and related taxa							
* * Kernia columnaris	CBS 159.66	MN991957					MN982416
* * Kernia geniculotricha	CBS 599.68	MN991956					MN982414
* * Kernia nitida	CBS 282.52	MN991955					MN982415
* * Kernia pachypleura	CBS 776.70	MN991958					MN982417
* * Lomentospora prolificans	CBS 114.90	MH862198					
	DTO 402-E9	MT316371					MT433464
* * Lophotrichus fimeti	CBS 129.78	MH861119					
* * Lophotrichus macrosporus	CBS 379.78	MH861152					
* * Microascus longirostris	CBS 196.61	LM652421					LM652634
* * Parascedosporium putredinis	CBS 108.10	MH854594					
	CBS 133438	MH866067					
* * Parascedosporium tectonae	CBS 118694	AM749735					
* * Petriella guttulata	CBS 362.61	MH858084					
* * Petriella setifera	CBS 385.87	AY882345					EU977491
* * Petriella sordida	CBS 144612	MK442608					MK442740
* * Petriellopsis africana	CBS 311.72	AJ888425					AJ889603
* * Scedosporium americanum	CBS 218.35	AM712309					MT813192
	DMic 165285	MT803031					MT813191
* * Scedosporium angusta	CBS 116914	KT008539					KT008468
	CBS 254.72	AY228114					KT008467
* * Scedosporium apiospermum	CBS 101719	KT008504					KT008486
	CBS 117399	KT008503					KT008485
	CBS 117405	KT008514					KT008483
	CBS 117411	KT008513					KT008484
	GZUIFR 21.835^*a*^	MZ469291					MZ488565
	GZUIFR 21.836^*a*^	MZ469292					MZ488566
* * Scedosporium aurantiacum	CBS 103.44	KT008559					KT008437
	CBS 117414	KT008558					KT008436
	CBS 117426	KT008560					KT008435
	GZUIFR 21.838^*a*^	MZ469294					MZ488568
	GZUIFR 21.839^*a*^	MZ469295					MZ488569
* * Scedosporium boydii	CBS 116898	KT008520					KT008458
	CBS 117390	KT008528					KT008465
	CBS 117392	KT008530					KT008466
	CBS 117417	KT008526					KT008464
	CBS 117432	KT008516					KT008456
* * Scedosporium cereisporum	FMR 12996	KJ599660					KJ599659
* * Scedosporium dehoogii	CBS 117387	KT008552					KT008494
	CBS 117393	KT008553					KT008495
	CBS 117406	KT163400					KT163401
	GZUIFR 21.837^*a*^	MZ469293					MZ488567
* * Scedosporium desertorum	CBS 489.72	MH860541					KT008438
* * Scedosporium ellipsoideum	CBS 418.73	AJ888426					AJ889595
* * Scedosporium fusoideum	CBS 106.53	AJ888428					AJ889601
* * Scedosporium haikouense	GZUIFR 21.833^*a*^	MZ469289					MZ488563
	GZUIFR 21.834^*a*^	MZ469290					MZ488564
* * Scedosporium hainanense	GZUIFR 21.827^*a*^	MZ469283					MZ488557
	GZUIFR 21.828^*a*^	MZ469284					MZ488558
	GZUIFR 21.829^*a*^	MZ469285					MZ488559
* * Scedosporium hunanense	GZUIFR 21.830^*a*^	MZ469286					MZ488560
	GZUIFR 21.831^*a*^	MZ469287					MZ488561
	GZUIFR 21.832^*a*^	MZ469288					MZ488562
* * Scedosporium minutisporum	CBS 100396	KT008555					KT008440
	CBS 116595	KT008557					KT008439
	CBS 116911	KT008556					KT008441
	FMR 4072	AJ888384					AJ889592
* * Scedosporium rarisporum	G79	KX790702					
* * Scedosporium sanyaense	EM 65901	KJ001005					
	EM 65901.2	KX790701					
* Scedosporium* sp.	GZUIFR 21.840^*a*^	MZ469296					MN541380
* * Scopulariopsis brevicaulis	MUCL 40726	LM652465					LM652672
* * Wardomyces anomalus	CBS 299.61	LN850992					LN851149
* * Wardomyces giganteus	CBS 746.69	LM652411					LN851150
* * Wardomyces humicola	CBS 369.62	LN850993					LN851151
* * Wardomyces inflatus	CBS 216.61	LM652496					LN851152

a Accession numbers for these strains generated from this study.
